# The Use of Compounds Derived from *Cannabis sativa* in the Treatment of Epilepsy, Painful Conditions, and Neuropsychiatric and Neurodegenerative Disorders

**DOI:** 10.3390/ijms25115749

**Published:** 2024-05-25

**Authors:** Anna Stasiłowicz-Krzemień, Wiktoria Nogalska, Zofia Maszewska, Mateusz Maleszka, Maria Dobroń, Agnieszka Szary, Aleksandra Kępa, Marcin Żarowski, Katarzyna Hojan, Malgorzata Lukowicz, Judyta Cielecka-Piontek

**Affiliations:** 1 Department of Pharmacognosy and Biomaterials, Faculty of Pharmacy, Poznan University of Medical Sciences, Rokietnicka 3, 60-806 Poznan, Poland; astasilowicz@ump.edu.pl (A.S.-K.);; 2 Department of Developmental Neurology, Poznan University of Medical Sciences, Przybyszewski 49, 60-355 Poznan, Poland; zarowski@ump.edu.pl; 3 Department of Occupational Therapy, Poznan University of Medical Sciences, Swięcickiego 6, 61-847 Poznan, Poland; khojan@ump.edu.pl; 4 Department of Rehabilitation, Greater Poland Cancer Centre, Garbary 15, 61-866 Poznan, Poland; 5 Department of Rehabilitation, Centre of Postgraduate Medical Education, Konarskiego 13, 05-400 Otwock, Poland; gosialukowicz@wp.pl; 6 Department of Pharmacology and Phytochemistry, Institute of Natural Fibres and Medicinal Plants, Wojska Polskiego 71b, 60-630 Poznan, Poland

**Keywords:** cannabis, *Cannabis sativa*, cannabidiol, tetrahydrocannabinol, neurological disorders

## Abstract

Neurological disorders present a wide range of symptoms and challenges in diagnosis and treatment. *Cannabis sativa*, with its diverse chemical composition, offers potential therapeutic benefits due to its anticonvulsive, analgesic, anti-inflammatory, and neuroprotective properties. Beyond cannabinoids, cannabis contains terpenes and polyphenols, which synergistically enhance its pharmacological effects. Various administration routes, including vaporization, oral ingestion, sublingual, and rectal, provide flexibility in treatment delivery. This review shows the therapeutic efficacy of cannabis in managing neurological disorders such as epilepsy, neurodegenerative diseases, neurodevelopmental disorders, psychiatric disorders, and painful pathologies. Drawing from surveys, patient studies, and clinical trials, it highlights the potential of cannabis in alleviating symptoms, slowing disease progression, and improving overall quality of life for patients. Understanding the diverse therapeutic mechanisms of cannabis can open up possibilities for using this plant for individual patient needs.

## 1. Introduction

Neurological disorders encompass a wide range of conditions with diverse etiologies. These disorders can be categorized into primary neurological disorders, including strokes, epilepsy, movement disorders, headaches, intervertebral disc disorders, lumbar and cervical arthrosis, polyneuropathy, Parkinson’s disease, and Alzheimer’s disease [1,2,3]. Neurodegeneration is a complex process characterized by the progressive loss of structure or function of neurons in the brain, leading to cognitive decline, motor dysfunction, and other neurological symptoms [4]. While neurological disorders share similarities with psychiatric disorders in terms of brain involvement and symptom overlap, they are distinct entities [5]. In the field of mental health, disorders such as depression, anxiety, and posttraumatic stress disorder (PTSD) represent significant areas of health service. These conditions extend beyond transient emotions, profoundly influencing cognitive, emotional, and behavioral domains. Despite advancements, the enduring impact of these disorders underscores the ongoing need for scholarly inquiry and clinical intervention. Around 280 million people globally grapple with this mental health condition [6]. Approximately 3.8% of the population, including 5% of adults and 5.7% of adults older than 60 years, experience depression. An estimated 34% of adults in the United States experience anxiety disorders at some point in their lifetime, contributing to notable distress and functional challenges [7]. PTSD, a psychiatric condition, emerges following exposure to actual or potential harm, including experiences of injury, death, or sexual assault, leading to significant functional and cognitive difficulties [8]. Neurodevelopmental disorders, such as autism spectrum disorder and attention-deficit/hyperactivity disorder (ADHD), have undergone diagnostic advancements over time [9,10]. ADHD is characterized by a persistent pattern of inattention, hyperactivity, and impulsivity [11]. Individuals with ADHD may exhibit difficulties in maintaining attention, controlling impulses, and regulating their activity levels, which can impact various aspects of their daily functioning. The prevalence of ADHD in children and adolescents is notably high, with approximately 7.6% of children aged 3 to 12 years and 5.6% of teenagers aged 12 to 18 years [12]. Research has shown that ADHD is associated with delays in cortical maturation, suggesting neuroanatomic differences in individuals with the disorder [13]. Moreover, ADHD has been linked to various comorbidities, including internet addiction, depression, anxiety, and sleep problems [14,15]. Another neurodevelopmental disorder is autism spectrum disorder, which is characterized by challenges in social communication, repetitive behaviors, and restricted interests [16]. Autism spectrum disorder encompasses a wide range of symptoms and severity levels, leading to a heterogeneous group of neurodevelopmental phenotypes diagnosed in more than 1% of children [17]. Functional movement disorders pose challenges in diagnosis due to their unique clinical presentation, requiring specific bedside signs and paraclinical tests for accurate differentiation from other neurological disorders [18,19]. Tourette syndrome is a neurodevelopmental disorder characterized by the presence of motor and vocal tics, which are sudden, repetitive, nonrhythmic movements or sounds [20]. Patients may experience a range of symptoms, including involuntary movements, vocalizations, and behavioral manifestations, which can vary in frequency and intensity over time. Tourette syndrome and persistent tic disorders may affect approximately 1.4 million people in the U.S., with an estimated prevalence of about 1 in 50 children aged 5–14 years [21]. Trigeminal neuralgia, characterized by severe facial pain, presents diagnostic complexities, especially with evolving headache disorder classification systems [22]. Neuropathic pain conditions, like painful neuropathy and central poststroke pain, underscore the intricate involvement of the somatosensory system in neurological disorders [23,24,25]. Epilepsy, defined by recurrent seizures, affects approximately 50 million individuals worldwide, solidifying its status as one of the most prevalent neurological conditions on a global scale [26]. The seizures result from abnormal electrical activity in the brain, leading to a wide range of symptoms, from momentary lapses in consciousness to convulsions [27]. Seizures in epilepsy can manifest in various forms, each with distinct characteristics that aid in their classification and diagnosis. The International League Against Epilepsy provides a framework for categorizing seizures and epilepsy syndromes based on their clinical presentation and underlying etiology [28]. Seizures can be broadly classified into different types, including focal seizures, generalized seizures, combined generalized and focal seizures, and unknown epilepsy groups. Focal seizures, also known as partial seizures, originate in a specific area of the brain and may present with motor or non-motor symptoms depending on the brain region involved [29]. Generalized seizures, on the other hand, involve both hemispheres of the brain from the onset and can present as tonic–clonic seizures, absence seizures, myoclonic seizures, or atonic seizures [30]. In addition to focal and generalized seizures, other seizure types are epileptic spasms and behavior arrest [31]. By accurately identifying and categorizing seizure types, healthcare providers can choose treatment to address the specific needs of individuals with epilepsy. Despite advancements in treatment, epilepsy can still pose significant difficulties to those affected, impacting daily activities, social interactions, and mental well-being [32]. Seizures that persist despite treatment with antiepileptic drugs present a significant challenge in epilepsy management. Drug-resistant epilepsy is described as the failure to achieve sustained seizure freedom despite adequate trials of at least two antiepileptic drugs used either in monotherapy or in combination [33]. Approximately one in three patients with epilepsy experience drug-resistant epilepsy [34,35]. Studies have explored the use of cannabis, especially CBD-enriched cannabis products, in both pediatric and adult patients with treatment-resistant epilepsy [36,37,38]. Gait disorders, such as ataxic gait, are prevalent in various neurological and musculoskeletal conditions, impacting individuals’ quality of life [39]. Given the diversity among neurological disorders and the complexities involved in their diagnosis and treatment, a crucial question arises: Is there a solution capable of effectively reducing symptoms and increasing the comfort and quality of life for patients afflicted with these conditions?

*Cannabis sativa*, a member of the *Cannabaceae* family, is a plant of immense scientific interest. It is renowned for its complex chemical composition, diverse physiological effects, and significant implications for pharmacology and medicine. *Cannabis sativa* possesses anticonvulsive, analgesic, anti-inflammatory, and neuroprotective effects [40,41]. Moreover, it has been reported to possess antioxidant, antibacterial, antifungal, anti-inflammatory, and anticancer properties [42]. *Cannabis sativa* has been found to contain over 500 chemical compounds, with more than 125 cannabinoids identified [43].

**Figure 1:**
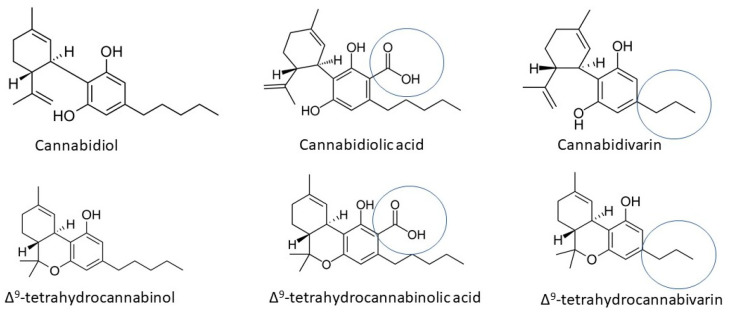
The structures of cannabinoids (cannabidiol and Δ^9^-tetrahydrocannabinol) in neutral, acidic, and varinic forms.

Cannabinoids can be broadly categorized into acidic, neutral, and varinic forms (Figure 1) [44]. Acidic cannabinoids, like cannabidiolic acid (CBDA) and tetrahydrocannabinolic acid (THCA), are the precursor forms found in the plant, which undergo decarboxylation to convert into their neutral counterparts upon heating or aging [45]. These acidic forms are typically found in raw cannabis plants and are less potent than their neutral counterparts. Neutral cannabinoids, such as cannabidiol (CBD) and Δ^9^-tetrahydrocannabinol (THC), are commonly known forms that interact with cannabinoid receptors in the human body, primarily CB1 and CB2 receptors, exerting various effects [46]. Varinic cannabinoids like tetrahydrocannabivarin (THCV) and cannabidivarin (CBDV) possess distinctive structures with specific alkyl side chains, setting them apart from other cannabinoids found in *Cannabis sativa* [47,48]. These structural differences influence their interactions with cannabinoid receptors and contribute to their pharmacological properties. 

Beyond cannabinoids, *Cannabis sativa* also contains non-cannabinoid compounds like terpenes and phenolic compounds, which contribute to its pharmacological properties [49]. These compounds work synergistically with cannabinoids, potentially enhancing their effects. 

**Figure 2:**
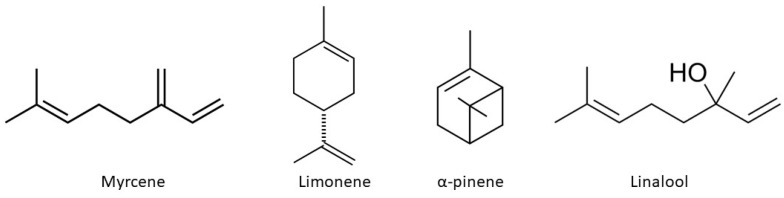
Chemical structures of chosen terpenes, myrcene, limonene, α-pinene, and linalool.

Terpenes contribute to cannabis’s distinct aroma and flavor profile (Figure 2). The terpene profile of *Cannabis sativa* can vary depending on factors such as genetics, growing conditions, and maturation stage [50]. Some common terpenes found in cannabis include myrcene, limonene, pinene, and linalool, each with unique aromatic properties and potential therapeutic effects. For example, myrcene is associated with sedative and analgesic effects, while limonene may have mood-enhancing properties [51,52]. Research has shown that terpenes can modulate the effects of cannabinoids by interacting with the endocannabinoid system and other molecular targets in the body [53]. Terpenes have been studied for their potential therapeutic benefits, including anti-inflammatory, analgesic, and anxiolytic properties [54]. Understanding the terpene profile of cannabis strains can help guide the selection of chemovars tailored to specific therapeutic needs [55].

**Figure 3:**
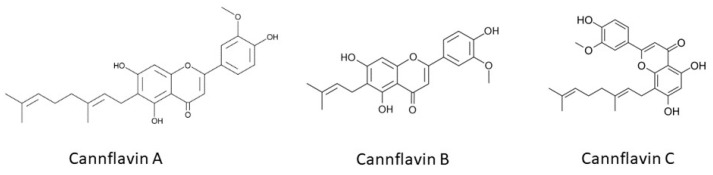
Chemical structures of cannflavins.

Polyphenols in cannabis include flavonoids, phenolic acids, and lignans, and they are known for their antioxidant, anti-inflammatory, and neuroprotective effects [54]. The flavonoid profile of cannabis extracts comprises flavones (apigenin and luteolin), flavonols (kaempferol and quercetin), aglycones, and glycosides [56]. Cannabis plants also have unique compounds, such as cannflavins, which are specific to the plant (Figure 3) [57]. These compounds, including cannflavin A, cannflavin B, and cannflavin C, have been studied for their diverse biological activities, particularly their anti-inflammatory and anticancer effects [58]. In addition to their anti-inflammatory effects, cannflavins have also demonstrated anticancer potential. Studies have highlighted the ability of cannflavins to inhibit the growth of cancer cells and induce apoptosis, suggesting their potential as novel anticancer agents [59]. 

While individual cannabinoids have gathered much attention for their medicinal properties, research suggests that the presence of other compounds in cannabis can modulate their effects and enhance therapeutic potential [51]. This synergistic effect (called the entourage effect) implies that whole-plant extracts containing cannabinoids, terpenes, and phenolic compounds may exhibit superior therapeutic efficacy compared to isolated cannabinoids or synthetic formulations [60]. Terpenes can influence the pharmacokinetics of cannabinoids by affecting their absorption, distribution, metabolism, and excretion [61]. This interplay between cannabis constituents contributes to the complex pharmacological effects observed in patients.

*Cannabis sativa* and its extracts can be administered to patients through various routes to achieve therapeutic effects. Vaporization of cannabis extracts has gained significant popularity due to its ability to deliver the active compounds of cannabis without the negative byproducts typically associated with smoking [62]. Unlike traditional smoking methods, which involve combustion and the generation of harmful substances like tar and carbon monoxide, vaporization heats the cannabis material to a temperature that releases its active ingredients as a vapor without causing combustion. This process offers several advantages beyond harm reduction. One notable advantage is the rapid onset of effects [63]. When cannabis is vaporized, the active compounds are absorbed into the bloodstream through the lungs. This rapid onset is particularly beneficial for individuals seeking immediate relief from symptoms such as pain or nausea. Oral administration of cannabis extracts, such as oils, capsules, or edibles, provides a convenient and discreet method for patients to administer cannabinoids [64,65]. This route allows for precise dosing, making it suitable for patients who require controlled and consistent intake of medication. However, oral ingestion also poses some challenges in terms of pharmacokinetics. Pharmacokinetic studies have shown that oral administration of cannabis extracts results in slower absorption and prolonged duration of action compared to inhalation [66]. Peak plasma concentrations typically occur 1 to 2 h after ingestion, with effects lasting for several hours or longer depending on the dose and individual metabolism [67]. When cannabinoids are consumed orally, they undergo first-pass metabolism in the liver before entering the bloodstream. Once THC enters the liver, it undergoes enzymatic conversion into 11-hydroxy-Δ^9^-tetrahydrocannabinol, which is known to be more potent and longer-lasting than THC itself [68]. Unlike other routes of administration, such as smoking or vaporization, where THC is rapidly absorbed into the bloodstream and reaches the brain almost immediately, oral ingestion results in a slower onset of effects due to the need for digestion and metabolism. Consequently, the experience of consuming cannabis orally can be characterized by a more pronounced and prolonged high compared to other methods of ingestion. Liquid cannabis extracts can be administered sublingually, where the extract is placed under the tongue for absorption into the bloodstream. This method allows for the rapid onset of effects due to the high vascularity of the sublingual mucosa [69,70]. Cannabis extracts can be formulated into topical products such as creams, lotions, or patches for application to the skin. This method is commonly used for localized relief of pain, inflammation, or skin conditions [71,72]. Cannabis extracts can also be formulated into suppositories for rectal or vaginal administration. This route allows for systemic absorption of cannabinoids and may be beneficial for patients who have difficulty with oral administration [73]. The literature about the pharmacokinetic parameters of cannabinoid rectal administration, especially THC, has demonstrated approximately doubled rates of bioavailability compared to oral routes [71,74]. This enhanced bioavailability can be attributed to several factors, including reduced hepatic enzyme metabolism, avoidance of acidic degradation, and increased absorption through rectal tissues. The rectum receives blood supply from three arteries, allowing efficient absorption of cannabinoids directly into the bloodstream, thus bypassing first-pass metabolism in the liver. The onset of activity following rectal administration is approximately 15 min, with effects lasting up to 12 h, making it a viable option for sustained symptom relief [75,76].

CBD can potentially have side effects, even without their immediate awareness. It has been associated with liver injury and can interfere with the efficacy of other medications [77,78]. CBD exhibits hepatoprotective effects by interacting with cannabinoid receptors (CB1 and CB2), peroxisome proliferator-activated receptors (PPARs), G protein-coupled receptor 55 (GPR55), and transient receptor potential channels (TRPs), thereby potentially reducing inflammation, oxidative stress, and apoptosis in liver diseases [79]. Moreover, in a randomized, double-blind, placebo-controlled study, THCV significantly decreased fasting plasma glucose and improved pancreatic β-cell function, adiponectin, and apolipoprotein A levels, suggesting its potential as a therapeutic agent for glycemic control in patients with type 2 diabetes [80]. Moreover, side effects of CBD may include changes in alertness, drowsiness, and gastrointestinal discomfort such as diarrhea and decreased appetite as well as alterations in mood such as irritability and agitation [81]. However, in small doses, CBD stimulates the central nervous system and improves concentration, which is visible in children with autism [82]. These effects typically subside when CBD usage is ceased or reduced. Cannabis, whether used as a plant or in extract form, can also have various side effects caused primarily by THC, the psychoactive compound in cannabis. These effects can vary depending on factors such as individual tolerance, method of consumption, dosage, and specific strain or product used. Common side effects associated with cannabis use include short-term memory impairment, impaired cognitive function, impaired motor skills, increased heart rate, dry mouth, red eyes, increased appetite, anxiety and paranoia, psychological dependence, respiratory issues (caused by smoking cannabis), risk of addiction, impaired judgment, and psychological effects [83].

Narrative reviews, while valuable in synthesizing information and providing a comprehensive overview of a topic, have inherent limitations that researchers should be aware of. These limitations include potential biases, subjectivity in data interpretation, lack of transparency in the review process, and the absence of a systematic approach to evidence synthesis [84]. Despite their usefulness in exploring complex phenomena and providing insights into diverse perspectives, narrative reviews may not always offer the same level of rigor and objectivity as systematic reviews or meta-analyses [85]. However, despite these limitations, narrative reviews are valuable in providing a holistic understanding of a topic by offering a broad overview and exploring diverse perspectives [86]. 

**Figure 4:**
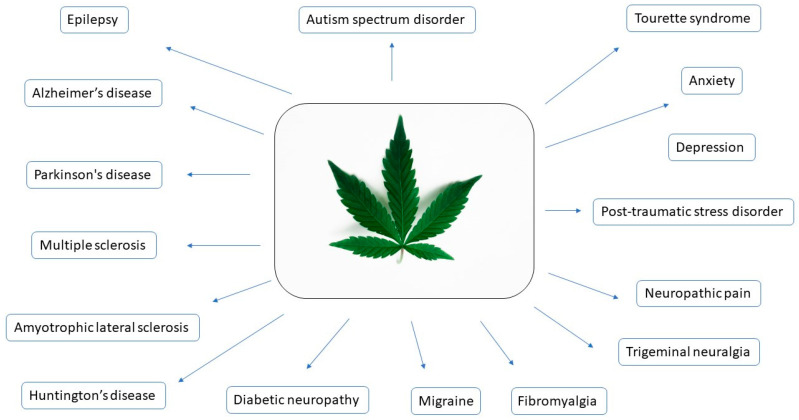
The potential of cannabis use in discussed neurological diseases of various etiologies.

The current review describes the use of cannabis and cannabis preparations for treating various neurological diseases, including epilepsy, neurodegenerative diseases, neurodevelopmental diseases, psychiatric disorders, and painful pathologies (Figure 4). This narrative review comprehensively examines the influence of cannabis and cannabinoids on the abovementioned diseases, elucidating both the molecular mechanisms of these conditions and the proposed mechanisms of cannabinoid activity. While the existing literature often focuses on individual diseases, this review consolidates and explores multiple perspectives on a wide range of diseases. Based on the data from surveys, patient studies, and clinical trials, the review sheds light on the therapeutic efficacy of cannabis in managing symptoms across these neurological disorders. It evaluates the outcomes observed in patients who have incorporated cannabis into their treatment regimens, providing insights into its potential benefits in symptom relief, disease progression, and overall quality of life. 

## 2. Neurological Disorders and Cannabis Studies

### 2.1. Epilepsy

Epilepsy is a neurological disorder identified by repetitive and unpredictable disruptions in the normal functioning of the brain, commonly referred to as seizures. It should be noted that epilepsy isn’t a singular disease but rather a range of disorders indicating a fundamental dysfunction in the brain, which may stem from diverse origins [87]. Epilepsy epidemiology encompasses various aspects of the occurrence of this condition in populations. In studies on the incidence of epilepsy, the cumulative rate was found to be 61.4 cases per 100,000 person-years, with a higher incidence in low/middle-income countries compared to high-income countries. The incidence and prevalence of epilepsy are slightly higher in men than in women. The epilepsy epidemic also shows cultural, socio-economic, and ethnic variations [88]. The molecular basis of epilepsy involves intricate mechanisms influencing neuronal function and excitability. Ion channel dysfunction is a prominent factor, characterized by mutations in genes encoding voltage-gated sodium channels (e.g., SCN1A, SCN2A), potassium channels (e.g., KCNQ2, KCNQ3), and calcium channels (e.g., CACNA1A), which disrupts normal neuronal excitability thresholds and propagation [89,90]. Furthermore, dysregulation of gamma-aminobutyric acid (GABA) signaling and glutamatergic signaling significantly impacts the balance between excitatory and inhibitory neurotransmission [91]. Alterations in GABA receptors (e.g., GABRA1, GABRG2) and transporters contribute to hyperexcitability [92], while abnormalities in glutamate receptors (e.g., NMDA receptors) and transporters exacerbate neuronal hyperexcitability and seizure generation [93]. Beyond neurotransmitter signaling, imbalances in other neurotransmitter systems, such as serotonin, dopamine, and acetylcholine, are implicated in epilepsy pathophysiology [94]. Additionally, alterations in proteins regulating neuronal excitability and synaptic plasticity, including neurotransmitter receptors and ion channels, further influence seizure susceptibility [91,95]. Moreover, neuroinflammation, characterized by the activation of microglia and the release of pro-inflammatory cytokines, can alter neuronal excitability and contribute to seizure progression [96]. Mitochondrial dysfunction, involving defects in energy metabolism and oxidative stress, also plays a role in epileptogenesis [97]. Epigenetic modifications, such as changes in DNA methylation, histone modifications, and non-coding RNA expression, further contribute to epilepsy susceptibility and seizure development by influencing gene expression patterns in the brain [98]. The retrospective study by Tzadok et al. in four pediatric centers in Israel examined CBD-enriched cannabis oil for treating intractable epilepsy in children [99]. Seventy-four patients, mostly under ten years old, with refractory epilepsy participated, receiving CBD-enriched cannabis oil after failed attempts with medication, a ketogenic diet, or vagal nerve stimulation. The CBD-enriched cannabis oil, containing 20% CBD and 1% THC, was supplied by licensed growers. Patients were divided into two dosage groups (1–10 mg/kg/d and 10–20 mg/kg/d) with a treatment period ranging from 3 to 12 months. Positive outcomes, including a reduction in seizure frequency (89%) and improved behavior, were reported in 44% of patients. Adverse events leading to CBD-enriched cannabis oil withdrawal in 5 cases included seizure aggravation (7%) and somnolence/fatigue (22%) [99]. The retrospective study by Porcari et al. aimed to assess the effectiveness of artisanal CBD-containing products in treating medically resistant epilepsy in 209 patients below 18 years old. Data were collected between 2006 and 2016 using the Synthetic Derivative at Vanderbilt University Medical Center [100]. The analysis focused on the impact of adding artisanal CBD products to the patient’s treatment regimen. The study considered factors such as seizure frequency, response to intervention, side effects, and changes in medications. A subgroup analysis of patients using both clobazam and CBD was conducted to assess potential interactions between these substances. Responses of patients using clobazam alone were identified for comparison. The results indicated that artisanal CBD was beneficial in treating medically resistant seizures, with a reported reduction in seizure frequency of at least 50% observed in 33% of the CBD group, 44% of the CBD + clobazam group, and 38% of the clobazam group. Minimal differences between groups suggested that CBD may provide comparable benefits regardless of clobazam use. For side effects, sedation was more frequent in the clobazam group (36%) compared to the CBD + clobazam group (7%) and the CBD group (0%) [100]. Pamplona et al.’s meta-analysis of 11 studies (including the two presented before) on CBD in pediatric epilepsy, spanning 2013 to 2017 and involving 670 patients, found that CBD significantly reduced seizure frequency, with 64% of patients showing improvement [101]. Studies with CBD-rich cannabis extracts (71%) reported more improvement than those with purified CBD (46%). Responders to a 50% or more reduction in seizure frequency were 39%, with no significant difference between treatments. The average daily CBD dose was 15.0 mg/kg, while purified CBD was 25.3 mg/kg, and CBD-rich cannabis extract was 6.0 mg/kg. Secondary health improvements, such as improvements in awareness, sleep quality, mood, behavior, language, and motor skills, were reported in 52% of patients. Adverse events occurred in 51%, with CBD-rich extracts (76%) having more mild events than purified CBD (33%). Severe adverse events were reported in 15% of patients, including appetite alteration, sleepiness, gastrointestinal disturbances, weight changes, fatigue, and nausea [101].

Dravet syndrome is a severe form of myoclonic epilepsy that begins in infancy and is associated with functional and morphological brain abnormalities [102]. Dravet syndrome is accompanied by coexisting conditions such as intellectual disability, sleep and gait disorders, and behavioral issues. It is rare and is diagnosed in 1 in 20,000 to 40,000 live births. This syndrome is caused by mutations in the SCN1A sodium channel gene, inherited in an autosomal dominant manner, affecting the majority of patients (80%). In a minority of cases, disorders occur in genes such as SCN9A, SCN2B, PCDH19, GABRG2, GABRA1, and STXBP1, with a frequency estimated at 15.9–18% of the population. 

Xu et al. summarized evidence on the direct and indirect mechanisms of action of sodium, potassium, and HCN channels in Dravet syndrome and understanding the application of CBD, whose antiepileptic effects enable modulation of these channels [103]. CBD has demonstrated neuroprotective effects in experimental animal models of Dravet syndrome as well as clinical data. It is suggested that CBD may restore the balance of excitatory/inhibitory neurotransmission by enhancing GABAergic/inhibitory transmission or reducing excitatory transmission. Therefore, it can be speculated that the action of CBD may result from its influence on multiple ion channels underlying Dravet syndrome channelopathies. Devinsky et al. conducted a study on the use of CBD in children and young adults with Dravet syndrome and drug-resistant seizures [104]. The study included 120 patients who received an oral solution of CBD at a dose of 20 mg/kg body weight per day or placebo in addition to standard antiepileptic treatment for 14 weeks. The use of the therapy showed a reduction in seizures and a decrease in their average frequency from 14.9 to 5.9. Patients in whom a minimum of 50% reduced the frequency of seizures amounted to 43%. Adverse effects observed during the study included diarrhea, vomiting, fatigue, fever, increased drowsiness, and abnormal results of liver indices. The use of CBD reduced the incidence of seizures but was associated with a higher rate of side effects.

**Figure 5:**
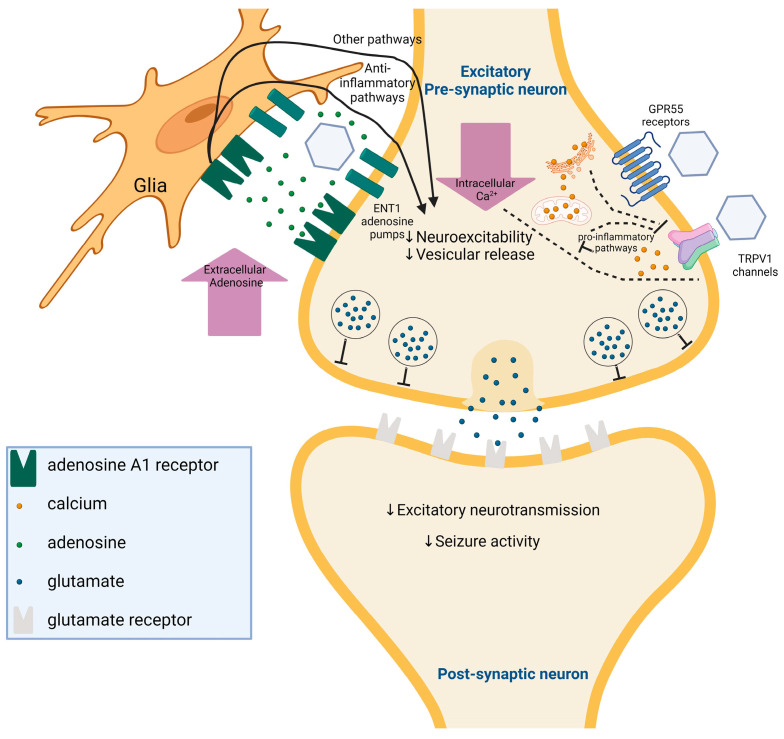
The possible mechanisms of action of CBD in epilepsy [105].

The mechanism of action of CBD in epilepsy involves its interaction with multiple molecular targets (Figure 5), including GPR55, TRPV1, and the adenosine system [105]. CBD has been shown to antagonize GPR55 receptor activation [106]. GPR55’s involvement in modulating neuronal excitability is underscored by its increased expression in the epileptic hippocampus [107]. Functional antagonism of GPR55 by CBD reduces neuronal excitability, contributing to its anticonvulsive effects. TRPV1 channels are expressed widely in the central nervous system and play a role in promoting neuronal depolarization [108]. CBD activates and rapidly desensitizes TRPV1 receptors, leading to a decrease in synaptic activity and neuronal firing rate [109,110]. Increased TRPV1 expression in epilepsy suggests its involvement in regulating cortical excitability [111,112,113]. The anticonvulsive action of CBD involves TRPV1, as demonstrated by CBD’s blunted response in TRPV1 knock-out mice [114]. Adenosine is an endogenous modulator of neuronal excitability and plays a crucial role in terminating seizures [115]. CBD enhances adenosine-mediated signaling by increasing extracellular adenosine levels through inhibition of adenosine reuptake. Adenosine exerts its anticonvulsant effects primarily through activation of A1 receptors, leading to presynaptic calcium influx inhibition and postsynaptic hyperpolarization [116]. Adenosine further fine-tunes neuromodulation by engaging in heterodimerization with other G-protein-coupled receptors [117]. This interaction extends adenosine’s influence across various neurotransmitter and neurotrophin systems. CBD’s modulation of adenosine transport contributes to its anticonvulsive properties, enhancing adenosine’s inhibitory effects on excitatory transmission. While the precise mechanism of action of CBD in humans is not fully understood, preclinical evidence strongly implicates GPR55 antagonism, TRPV1 desensitization, and adenosine modulation in CBD’s anticonvulsive effects. These mechanisms collectively reduce neuronal excitability and modulate neurotransmitter release, leading to the suppression of seizure activity Further research is needed to elucidate additional targets and pathways involved in CBD’s therapeutic action against epilepsy.

### 2.2. Neurodegenerative Diseases

#### 2.2.1. Parkinson’s Disease

Parkinson’s disease is a multifactorial degenerative disease. It leads to tremors, gait rigidity, motor slowing, and postural instability as well as non-motor symptoms such as sleep disturbances, depression, cognitive deficits, and neuropsychiatric symptoms [118,119]. Its prevalence increases with age, and in North America, it is 572 per 100,000 people over the age of 45 [120]. Parkinson’s disease is characterized by the gradual loss of dopaminergic neurons in the substantia nigra pars compacta region of the brain [121]. The aggregation of misfolded alpha-synuclein protein and Lewy bodies within neurons is a feature of Parkinson’s disease [122]. These aggregates disrupt cellular function, leading to neuronal dysfunction and eventual death. Additionally, mitochondrial dysfunction exacerbates the condition, causing increased oxidative stress and energy deficits, further contributing to neuronal damage [123]. Oxidative stress, arising from an imbalance between reactive oxygen species (ROS) production and detoxification, inflicts cellular damage through lipid peroxidation, protein oxidation, and DNA damage, accelerating neuronal degeneration [124]. Moreover, neuroinflammation, driven by activated microglia releasing pro-inflammatory cytokines, exacerbates neuronal injury [125]. The ubiquitin-proteasome system and autophagy-lysosomal pathway lead to the accumulation of misfolded proteins like alpha-synuclein, enhancing neuronal toxicity [126,127]. Genetic mutations, particularly in genes such as SNCA, LRRK2, Parkin, PINK1, and DJ-1, contribute to Parkinson’s disease [128]. Environmental toxins like pesticides and heavy metals amplify Parkinson’s disease risk by inducing oxidative stress, mitochondrial dysfunction, and alpha-synuclein aggregation [129]. 

Sousa et al. conducted a study involving 15 individuals with Parkinson’s disease, averaging 67 years old [130]. Participants were administered a combination of CBD and THC via vaporization. A comparison between those receiving CBD/THC treatment and those not revealed that CBD/THC recipients exhibited poorer cognitive ability, as measured by the MoCA test, and experienced non-motor symptoms of Parkinson’s disease. However, despite these findings, benefits such as pain reduction, anxiety relief, and improved sleep quality were observed. Reported side effects included drowsiness and cognitive difficulties associated with cannabis products. In a study examining cannabis use patterns among nearly 400 Parkinson’s disease patients, it was found that 25% of them used cannabis, with 45.9% reporting benefits [131]. Notably, cannabinoids were most effective in alleviating bradykinesia, a key symptom of Parkinson’s disease. Relief from unintentional involuntary movements was reported by 14% of patients. Additionally, in a short pilot study, the cannabinoid receptor agonist nabilone significantly reduced levodopa-induced dyskinesia, a common treatment for Parkinson’s symptoms [132]. A study found that among 84 patients who smoked marijuana, 31% reported improvement in resting tremor, and 45% reported a decrease in motor slowing [133]. In contrast, among 22 Parkinson’s patients surveyed who smoked cannabis (0.5 g of cannabis), 30% reported subjective improvements in tremor, rigidity, motor slowing, pain, and sleep 30 min after smoking cannabis [134]. In another small randomized, double-blind, placebo-controlled trial, subjects were given the CBD agent in doses of 75 mg or 300 mg/day [133]. Among the respondents, there was no change in the Unified Parkinson’s Disease Rating Scale total score or any other subscales. There was, however, an improvement in the total Parkinson’s Disease Questionnaire-39. Scores of partial activities of daily living also improved for the group receiving CBD at a dose of 300 mg/day.

Preclinical study results suggest mechanisms that might be underlying the potential therapeutic effects of cannabis in Parkinson’s disease. CB1 agonists have been shown to inhibit basal ganglia dopamine release, thus potentially exacerbating motor symptoms. However, some studies indicate that certain CB1 agonists may ameliorate motor impairment through non-dopaminergic pathways, including interactions with adenosine A2A and serotonin (5-HT) receptors [135,136,137,138,139]. Conversely, studies on CB1 antagonists have more consistently demonstrated improvements in motor symptoms [140,141,142,143], possibly through mechanisms such as enhanced striatal glutamate release [142,143,144]. The involvement of the endocannabinoid system in levodopa-induced dyskinesias is contentious, with conflicting findings in experimental Parkinson’s disease models and chronic levodopa treatment [145,146,147]. Nevertheless, both CB1 agonists and antagonists have shown potential as antidyskinetic agents in preclinical studies [135,141,148]. The antidyskinetic effects of CB1 agonists may involve normalization of cAMP/PKA signaling and increased DARPP-32 phosphorylation, although higher doses may lead to motor impairment [148,149,150,151,152]. Interestingly, FAAH inhibitors, which elevate endocannabinoid levels, only displayed antidyskinetic properties when combined with a TRPV1 receptor antagonist, suggesting opposing roles of CB1 and TRPV1 receptors in levodopa-induced dyskinesias regulation [150]. Furthermore, certain cannabinoids like anandamide may reduce levodopa-induced dyskinesias by activating PPAR-γ, while the endogenous lipid ligand for PPAR-α, oleoylethanolamide, showed beneficial effects attributed to TRPV1 receptor blockade rather than PPAR-α activation [153,154].

#### 2.2.2. Alzheimer’s Disease

Alzheimer’s disease represents a multifaceted disorder involving a combination of genetic mutations, environmental factors, and the aging process [155]. Alzheimer’s disease’s symptoms evolve with its progression. Initially, patients commonly experience episodic short-term memory loss, followed by difficulties in problem-solving, judgment, and executive functioning. Language and visuospatial skills may also deteriorate. In later stages, neuropsychiatric symptoms like apathy, agitation, and psychosis may arise, along with motor impairments [156]. Alzheimer’s disease encompasses both sporadic and familial forms, with genetic mutations in genes like amyloid precursor protein (APP) and presenilins (PSEN1 and PSEN2) implicated in family cases [157]. However, the exact etiology of sporadic Alzheimer’s disease remains elusive, influenced by a complex interplay of genetic, epigenetic, environmental, and lifestyle factors. Globally, Alzheimer’s disease affects over 50 million individuals, a number projected to double in the coming decades due to increasing human lifespan and the associated rise in Alzheimer’s disease risk with aging [155]. This epidemiological trend underscores the urgent need to address Alzheimer’s disease as a significant public health concern. Understanding the multifactorial etiology and epidemiological trends of Alzheimer’s disease is crucial for developing effective interventions to mitigate its impact on individuals and societies worldwide [155].

A double-blind RCT investigated nabilone’s efficacy and safety for agitation in moderate-to-severe Alzheimer’s disease, enrolling 39 patients, with 38 included in the analysis [158]. The study employed a 14-week crossover design with a 1-week single-blind placebo phase preceding each treatment phase. Nabilone oral dosing ranged from 0.25 mg to a maximum of 2 mg/day, with adjustments based on tolerability. Safety assessments included monitoring for TEAEs, SAEs, and vital signs. Nabilone showed efficacy in reducing agitation (CMAI), with a medium effect size (Cohen’s d = 0.52). Secondary outcomes favored nabilone, including neuropsychiatric symptoms, caregiver distress, cognition, and global change. Despite efficacy, higher rates of treatment-emergent adverse events (TEAEs), notably sedation, were associated with nabilone, though dose reduction often ameliorated these effects. Another study was part of a Phase II, repeated crossover, double-blind RCT evaluating the efficacy and safety of two different doses (0.75 and 1.5 mg twice daily) of oral THC in addressing dementia-related neuropsychiatric symptoms (NPS) [159]. Conducted between September 2011 and December 2013, the trial involved 22 participants at the Radboudumc Alzheimer Centre and the Vincent van Gogh Institute for Psychiatry in the Netherlands. Mobility assessments focused on the effects of the highest THC dose of 1.5 mg, administered twice daily. Notably, the study found significant increases in dynamic balance, stride length, and gait velocity after THC administration compared to placebo. However, adverse events such as dizziness, somnolence, balance disorders, and falls were reported, with more falls occurring during placebo treatment than THC, particularly at the lower THC dose [159]. 

The mechanism of cannabinoid activity in Alzheimer’s disease is multifaceted, involving the modulation of various cellular pathways. By interacting with CB receptors, cannabinoids (CBD, THC) exert regulatory effects on neurotransmitter release, synaptic plasticity, and neuroinflammatory responses [160]. CBD’s mechanisms in Alzheimer’s disease are better elucidated than for THC. CBD down-regulates glycogen synthase kinase 3β (GSK-3β), a key enzyme involved in tau phosphorylation [161]. Additionally, CBD modulates transient receptor potential vallinoid 1 (TRPV1) signaling and activates the phosphatidylinositol 3-kinase/Akt kinase (PI3K/Akt) pathway, contributing to its neuroprotective effects [162]. Furthermore, CBD promotes the degradation of APP and reduces the expression of enzymes involved in Aβ production [163]. Moreover, CBD increases cell survival, reduces lipid peroxidation, and ROS production [162]. CBD also attenuates nitric oxide production by inhibiting phosphorylated p38 mitogen-activated protein kinase (p38 MAPK) and transcription factor nuclear factor-κB (NF-κB) [161]. Additionally, CBD down-regulates the expression of β-secretase 1 (BACE1), PS1, and PS2 genes, which encode enzymes involved in Aβ production [162]. CBD’s protective effects extend to mitochondrial function. It rescues iron-induced apoptosis, restores levels of hippocampal dynamin-related protein 1, a mitochondrial fission protein, and reverses increased mitochondrial ferritin and altered mitochondrial epigenetic modulation, ultimately leading to increased neuronal survival [164,165]. In addition to CBD, other minor cannabinoids such as CBN, CBG, and CBC exhibit anti-inflammatory effects and promote the degradation of Aβ aggregates [166,167].

#### 2.2.3. Multiple Sclerosis

Multiple sclerosis is a complex autoimmune disorder of the central nervous system characterized by chronic inflammation, demyelination, and neurodegeneration [168]. Its pathogenesis is multifactorial, involving intricate interactions between genetic predisposition, environmental triggers, and dysregulated immune responses [169]. Genetic susceptibility is evident, with certain alleles, particularly within the major histocompatibility complex region such as HLA-DRB1, exhibiting strong associations with multiple sclerosis risk [170,171]. Immune dysregulation lies at the core of multiple sclerosis pathology, where autoreactive T cells, activated by environmental cues or viral infections, infiltrate the central nervous system parenchyma and target myelin antigens, instigating a cascade of pro-inflammatory responses [172]. The clinical course varies, usually beginning with reversible episodes of neurological deficits in the third or fourth decade of life. Later, between the sixth and seventh decades of life, it progresses to a disease characterized by permanent and irreversible neurological deterioration [173]. This demyelinating disease causes significant disturbances in the transmission of nerve signals between the brain and spinal cord, leading to damage to the myelin sheath [174]. Characteristic symptoms include spasticity, muscle spasms, tremors, bladder dysfunction, neuropathic pain, dysarthria, and some intellectual problems such as memory impairment [175].

Haddad et al. evaluated the efficacy of “nabiximols” oral mucosal spray, oral dronabinol, and oral forms of nabilone cannabis for the treatment of spasticity, pain, tremors, bladder function, sleep disturbances, quality of life, disability, and progression of disability associated with multiple sclerosis. Each dose of oral mucosa spray contains 2.7 mg, 2.5 mg, and 0.04 g of THC, CBD, and ethanol, respectively [174]. The recommended maximum daily dose is 12 sprays with an interval of at least 15 min between each administration of the drug. Nabiximols has been tested in several clinical trials in multiple sclerosis patients with various symptoms such as stiffness, pain, tremors, and bladder function [174]. Most studies have shown that nabiximols have a good effect on the symptoms mentioned above [174]. Filippini et al. conducted clinical trials with 3763 subjects aged between 18 and 60 years, where 2290 received a spray containing a 1:1 combination of THC and CBD, synthetic THC mimics, oral THC extract, or inhaled herbal cannabis. These were compared against a placebo group [176]. The duration of the study ranged from 3 to 48 weeks. Results indicate a significant reduction in the severity of MS-induced spasticity with nabiximols compared to the placebo group. Additionally, 67% of subjects reported experiencing a substantial decrease in MS-related discomfort [177]. Rainka et al. conducted a study involving 141 mainly female patients aged 51 to 70, using cannabinoids for chronic pain (80%) and/or spasticity (38%) [177]. The most common preparation was tincture, often supplemented with THC to CBD in a 20:1 ratio for breakthrough symptoms. The study noted a significant reduction in opioid use, with 22% discontinuing and 32% reducing opioid intake in favor of cannabis products. Average daily morphine milligram equivalents decreased from 51 mg to 40 mg. Patients reported subjective improvements: 72% experienced pain relief, 48% reported reduced muscle spasticity, and 40% improved sleep. Lesser improvements were noted in gait (11%), anxiety (11%), and quality of life (7%). According to the MUSEC study, which involved 279 participants (144 used medication, 135 placebo), the group received an oral cannabis extract [178]. During two weeks, patients received an escalating dose of 5 mg to a maximum of 25 mg of THC per day and a 10-week maintenance treatment phase. After the 12-week study, the results showed that the degree of muscle stiffness decreased almost twofold compared to the placebo group. In addition, side effects in participants treated with cannabis products were consistent with known side effects of cannabinoids and no new ones were observed. Cannabis products have been shown to significantly reduce spasticity and pain symptoms and improve quality of life among multiple sclerosis patients. A reduced frequency of opioid use, and consequently reduced opioid side effects, was noted among the study groups.

Dysregulated immune responses play a critical role in the pathogenesis of multiple sclerosis [179]. In multiple sclerosis, activated immune cells, including T cells and microglia, infiltrate the CNS and initiate an inflammatory cascade [180]. This leads to demyelination, axonal damage, and neuronal loss. Cannabinoids inhibit T-cell activation and proliferation, reduce the secretion of pro-inflammatory cytokines (e.g., TNF-α, IFN-γ), promote the apoptosis of activated immune cells, suppress microglial activation and proliferation, and promote the polarization of microglia/macrophages towards an anti-inflammatory phenotype [181,182,183]. They exert anti-inflammatory effects by modulating immune cell function [184]. Cannabinoids also reduce the expression of adhesion molecules and chemokines, thereby attenuating immune cell recruitment to the CNS [185,186]. This anti-inflammatory activity contributes to the mitigation of neuroinflammatory processes associated with multiple sclerosis pathology. Oligodendrocyte dysfunction and demyelination are central features of multiple sclerosis pathology [187]. Cannabinoids have been shown to promote remyelination. They stimulate the proliferation and differentiation of oligodendrocyte precursor cells and enhance the production of myelin proteins, facilitating the repair of damaged myelin sheaths [188]. Additionally, cannabinoids exhibit neuroprotective properties by reducing excitotoxicity, oxidative stress, and apoptosis [189,190]. They modulate neuronal excitability by regulating ion channels and neurotransmitter release, thereby preserving neuronal integrity and function. 

#### 2.2.4. Amyotrophic Lateral Sclerosis

Amyotrophic lateral sclerosis is a severe and progressive neuromuscular disorder that results in the degeneration of the motor neurons in the cortex, brain stem, and spinal cord [191]. Typically, symptoms like spasticity, hyperreflexia, and weakness emerge during the fifth or sixth decade of life. The disease progresses slowly, and its diagnosis relies on a comprehensive assessment of clinical symptoms alongside additional diagnostic tests. Determining the affliction involves excluding other potential disease [192]. Amyotrophic lateral sclerosis is an uncommon condition, manifesting at a rate of 1–2 cases per 100,000 people, and the incidence varies between 4–6 cases per 100,000. It is more prevalent in males, with a male-to-female ratio of 1.5:1. Amyotrophic lateral sclerosis is globally distributed with a comparable frequency. However, there is a slightly elevated incidence in the Western Pacific regions, specifically on Guam island and the Kii Peninsula in Japan, which is associated with the existence of familial forms of ALS in those geographical areas [193].

In a multicenter, double-blind, randomized, placebo-controlled, phase 2 trial assessing the safety and efficacy of nabiximols on spasticity symptoms in patients with motor neuron disease (CANALS), 60 participants were enrolled between 19 January 2013, and 15 December 2014 [194]. The final analysis included 59 participants, with 29 in the nabiximols group and 30 in the placebo group. Participants were allocated in a 1:1 (placebo:treatment) ratio to nabiximols. The primary endpoint was the change in the score on the Modified Ashworth Scale, evaluated at baseline after six weeks. Modified Ashworth Scale scores demonstrated an improvement by a mean of 0.11 (SD 0.48) in the nabiximols group while deteriorating by a mean of 0.16 (0.47) in the placebo group. Nabiximols demonstrated a beneficial impact on spasticity symptoms in individuals with motor neuron disease, exhibiting a satisfactory safety and tolerability profile [8]. Nabiximols was well tolerated, with no participant withdrawals during the double-blind phase of the study. Moreover, no adverse effects were reported.

#### 2.2.5. Huntington’s Disease

Huntington’s disease is an autosomal dominant condition that falls under the category of polyglutamine (polyQ) disorders, where it has one of the highest prevalence worldwide, right next to spinocerebellar ataxia type 3 [195]. The expansion of a CAG trinucleotide causes Huntington’s disease to repeat in the first exon of the Huntington gene (HTT), leading to the encoding of a mutant huntingtin (mHTT) protein [196,197]. That condition results in neurodegeneration, presenting symptoms such as movement disorders, dementia, and behavioral or psychiatric manifestations depending on the repeat range length of CAG. The normal number of repeats lies between 10 to 35. The greater the number of repeats, the higher the chance of expressing the condition, usually falling in the range of 40 to 55 [198,199,200]. Huntington’s disease does not show a preference for a specific gender, and symptoms generally occur in any age group. However, it most commonly affects individuals between the ages of 33 and 44. This condition occurs with varying frequencies worldwide, with the highest number of cases reported in Europe, affecting approximately 45,000 individuals, and in the USA, where the incidence is around 30,000 [198].

In a 21-day study by Aguareles et al., the administration of VCE-003.2, a derivative of CBG, at a concentration of 500 nM exhibited a pro-neurogenic effect in vitro [201]. The investigation utilized the mouse ES cell line R1 during neural differentiation, revealing an augmentation in CTIP2-positive cells and doublecortin immunoreactivity. Additionally, the study employed neuralized mouse embryonic teratocarcinoma P19 cells, wherein VCE-003.2 facilitated neuronal-like differentiation, confirmed by CTIP2 reporter activation. Furthermore, in a P19 neurosphere formation assay, VCE-003.2 prompted the development of larger neurospheres compared to the control group. These findings underscore the potential of VCE-003.2 to boost neural stem cell differentiation and substantiate its pro-neurogenic action. The study by Valdeolivas et al. aimed to explore the neuroprotective effects of Cannabigerol (CBG) in mice subjected to 3NP-induced toxicity, mimicking aspects of Huntington’s disease pathology, including hindlimb clasping, dystonia, and altered locomotor activity [202]. CBG administration ameliorated motor deficits, particularly hindlimb clasping, and dystonia, with no significant impact on truncal dystonia. In the striatal parenchyma of 3NP-treated mice, a marked reduction in Nissl-stained cells indicated neuronal death. Immunohistochemistry confirmed reduced NeuN-labeled neurons, accompanied by increased GFAP and Iba-1 immunostaining, indicative of astrogliosis and reactive microgliosis. CBG prevented neuronal death and 3NP-induced neuronal loss but showed modest effects on glial activation. mRNA expression analysis revealed upregulation of proinflammatory markers (COX-2, iNOS, TNF-α, and IL-6) in 3NP-lesioned mice. CBG significantly attenuated the upregulation of these markers, suggesting anti-inflammatory activity specifically on activated microglia. Biochemical analysis of oxidative stress markers in the striatum of 3NP-lesioned mice indicated reduced catalase and SOD activities, along with decreased GSH levels. CBG treatment restored antioxidant enzyme activities and GSH levels, demonstrating its neuroprotective potential against striatal damage induced by 3NP. A specific Huntington’s disease array analysis identified Cd44 and Sgk1 as significantly upregulated genes in 3NP mice. CBG treatment led to the downregulation of Cd44 and Sgk1, suggesting potential normalization of gene expression related to Huntington’s disease physiopathology. CBG and its derivatives exhibit significant anti-inflammatory effects in Huntington’s disease models, being particularly effective in aspects such as gene expression, alleviation of motor symptoms, reduction of microglial activation, and suppression of the inflammatory response. However, these results are still in the experimental stage, and further research is needed to assess the full neuroprotective potential of these phytocannabinoids and understand their mechanisms of action in the context of specific neurodegenerative diseases like Huntington’s disease [203]. 

### 2.3. Neurodevelopmental Disorders 

Neurodevelopmental disorders have a heterogeneous etiology, and their hallmarks are an inability to achieve cognitive, emotional, and motor developmental milestones, with consequent impairments in cognitive function, communication, adaptive behavior, and psychomotor skills. Neurodevelopmental disorders affect >3% of children worldwide [204]. An example of such a disorder is autism spectrum disorder, which is a common neurodevelopmental disorder characterized by difficulties in social communication and limited and repetitive interests [205]. Children with this disease often exhibit symptoms such as aggression, hyperactivity, and anxiety [206]. Worldwide, the prevalence oscillates between 0.08 and 9.3%, while in European countries, it is between 0.42 and 3.13% [207]. The treatment is complicated, as there are often concomitant diseases—behavioral or psychiatric—along with it. The majority (70%) of children and adolescents with autism spectrum disorder display aggressive behavior, and more than 25% of them also display self-injurious behavior. The standard treatment in Europe is either aripiprazole or risperidone, but their use, either alone or in combination, does not always have optimal efficacy [208].

#### 2.3.1. Attention Deficit-Hyperactivity Disorder

ADHD is a recognized psychiatric condition that significantly impacts children’s functional abilities. Children affected by this disease manifest characteristic tendencies towards an inappropriately advanced level of inattention, excessive arousal, or impulsivity [209]. The etiology of ADHD involves a complex interplay of genetic and environmental factors, with high heritability evidenced by greater concordance in monozygotic twins and increased sibling risk, along with potential prenatal influences such as viral infections, smoking, nutritional deficiencies, and alcohol exposure. Neuroimaging studies did not identify a cause; nevertheless, there is suspicion of reduced density of dopaminergic receptors in the frontal lobes as well as a potential involvement of noradrenergic receptors in the development of ADHD [210]. Various attention deficit disorder subtypes exhibit differing prevalence rates, with the inattentive subtype at 18.3%, hyperactive/impulsive at 8.3%, and combined at 70%. The inattentive subtype is more frequent in females, contributing to a 2:1 male-to-female ratio overall. ADHD affects 3–6% of adults and is notably prevalent in childhood, with potentially higher rates in the United States compared to other developed countries [209].

An EMA-C study, conducted over six weeks at King’s College London, was a double-blind, randomized, placebo-controlled trial investigating the efficacy of nabiximols in adults with ADHD. Nabiximols was administered comprising a 1:1 ratio of delta-THC to CBD. All subjects completed a two-week titration phase, followed by continuation at the determined optimal dose. The maximum dosage allowed for the study was 14 sprays daily. Although the primary outcome, cognitive performance assessed via the QbTest, did not demonstrate significant differences between nabiximols and placebo groups (F1, 28 = 2.04, *p* = 0.16), there were indications of potential benefits, with an estimated score reduction of 0.17 (95% CI 0.40 to 0.07) in the active group. Notably, minor adverse events such as light-headedness and diarrhea were reported in the active group, alongside two serious adverse events. One participant taking the active medication experienced sudden onset muscular seizures/spasms, possibly linked to the medication, while another participant on the placebo reported increased heart rate, chest tightness, and breathing difficulties, although no specific cause was identified. Despite these promising trends in ADHD symptom alleviation with nabiximols, vigilant monitoring remains imperative [211]. Another study investigated ADHD and cannabis effects on inhibitory control using MTA longitudinal data in 88 participants [212]. Cannabis use was self-reported, with 36 users and 52 non-users. Participants completed an fMRI Go/NoGo task with 192 trials across four runs. ADHD individuals showed more commission errors and cortical deactivation during inhibition, particularly in the right frontostriatal network. Subcortical regions like the right caudate exhibited reduced activation. An interaction effect between ADHD and cannabis was observed in the right hippocampus and cerebellar vermis. Post-hoc analyses confirmed these findings. Despite no significant cannabis impact on inhibition, the study highlights the intricate relationship between ADHD, cannabis, and inhibitory control [212].

The potential therapeutic effects of cannabinoids in ADHD are not fully understood. One proposed mechanism is the enhancement of dopaminergic transmission, similar to how stimulants alleviate ADHD symptoms [213,214,215,216]. However, evidence regarding the consistent enhancement of dopamine following cannabis use is inconclusive [217,218]. Other mechanisms may also be possible. The variability in the biochemical composition of cannabis makes elucidating specific mechanisms challenging. Some studies suggest that products high in THC or containing both THC and CBD may alleviate ADHD symptoms, but the optimal CBD:THC ratio remains uncertain and should be further studied [211,219,220]. 

#### 2.3.2. Autism Spectrum Disorder

Patients with autism spectrum disorder are characterized by problems with interpersonal, verbal, and nonverbal communication and restricted and repetitive patterns of behavior and actions [221]. Individuals with this disorder have difficulties in social interactions and verbal or nonverbal communication. They do not show language delay, and their cognitive development is not marked by general retardation but by specific impairments in certain areas, such as executive functions. The clinical picture is very heterogeneous, depending on age and psychiatric comorbidities. Screening, diagnosis, and specialized treatment are not easy due to the variety of clinical manifestations. It is often diagnosed late, on average at age 11, and, in some cases, even in adulthood, which has a significant impact on the risk of depression and poor quality of life [222]. The etiology has been linked to various genetic, neurological, and environmental factors, but the exact underlying mechanisms are poorly understood. 

Barchel et al. conducted a prospective study in 2018 with 53 participants between the ages of 4 and 22 [206]. Patients in the study were treated orally with CBD oil for 30–588 days. The cannabinoid oil solution was prepared at a concentration of 30% and a 1:20 ratio of CBD and THC. The recommended daily dose of CBD was 16 mg/kg (maximal daily dose 600 mg), and for THC, it was a daily dose of 0.8 mg/kg (maximal daily dose of 40 mg). Four symptoms of autism spectrum disorder comorbidities were assessed: hyperactivity symptoms, sleep problems, self-injury, and anxiety. Parents or caregivers, during follow-up interviews, reported a reduction in aggressive behavior and self-injury in 67.6% of those interviewed but a worsening in 8.8%. In 68.4%, hyperarousal symptoms improved and worsened in 2.6%, while they did not change in 28.9%. Sleep problems improved by as much as 71.4% while worsening by 4.7%. In 47.1% of the subjects, there was an improvement in anxiety and a worsening in 23.5%. The main reported side effects were drowsiness and loss of appetite. Aran et al. conducted a retrospective study in 2019 to evaluate the tolerability and efficacy of CBD-rich cannabis in 60 participants between the ages of 5 and 18 with autism spectrum disorder and severe behavioral problems. Patients were treated with CBD oil for 13 months [223]. The study found that 61% of participants experienced improvements in aggressive behavior. In addition to this, improvement in anxiety occurred in 39%, while improvement in communication occurred in 47%. One girl who took higher doses of THC experienced a transient serious psychotic event that required antipsychotic medication. In addition, reported side effects include sleep disturbances (14%), loss of appetite (9%), and irritability (9%). Also, in 2019, Fleury-Teixeira et al. conducted an observational study analyzing the effects of a *Cannabis sativa* extract containing a 75/1 CBD/THC ratio administered to a group of 18 patients with autism spectrum disorder [224]. Participants included 11 patients with no history of epilepsy, two previously diagnosed epileptics but without seizures for more than a year, and five currently diagnosed epileptics who had epileptic seizures in the month before *Cannabis sativa* treatment. The administration schedule included two daily doses, one in the morning and one in the evening. Three patients (one female and two males) decided to suspend treatment before the end of the first month due to adverse reactions. In two of them, the worsening of symptoms may have been the result of a simultaneous and unsupervised attempt to remove or reduce the dose of antipsychotics. Three patients may have experienced adverse effects as a result of interactions of prescribed cannabinoids with two other psychiatric medications. Of the remaining 15 patients (6–17 years old) who took CBD oil for 30–588 days, only one showed no improvement in autism spectrum symptoms. The most marked improvements were observed in sleep and behavioral disorders as well as in motor development, communication, social interaction, and cognitive performance. Side effects were mostly mild and included drowsiness, irritability, diarrhea, increased appetite, conjunctival congestion, and hyperthermia. Bar-lev Schleider et al. performed a retrospective study [225], during which 188 patients with autism spectrum disorder started treatment. The majority of patients were male (81.9%). The mean age of the patients was 12.9 ± 7.0 years. Of this subgroup, 60.0% were evaluated; 30.1% reported significant improvement, and 8.6% reported no change. After one month, 4.2% of patients discontinued treatment, and after six months, this percentage increased to 8.3%. The results showed that 25.2% of patients reported at least one side effect: anxiety (6.6%), drowsiness (3.2%), psychoactive symptoms (3.2%), increased appetite (3.2%), digestive disturbances (3.2%), dry mouth (2.2%) and lack of appetite (2.2%) [208]. CBD shows significant potential to safely alleviate many of the symptoms affecting children and adolescents on the autism spectrum. CBD is the most promising therapeutic cannabinoid for children and adolescents due to its safety profile, mild side effects, and broad-spectrum effects, including CBD’s anti-anxiety, antipsychotic, and neuroprotective properties [226]. 

Rett syndrome is another neurodevelopmental disorder coupled to the X chromosome. It is caused in 90% of cases by a mutation of the MECP2 gene [227]. In this disorder, there is a regression of previously acquired skills after a period of typical development. Rett syndrome can manifest itself in several symptoms, including head growth retardation, epilepsy, gait abnormalities, loss of speech, and breathing abnormalities. This is one of the most common causes of mental disability in women, with an incidence of 1 in 10,000 to 15,000 [228]. 

Desnous et al. [229] conducted a retrospective study in 2022 among 46 patients with Rett syndrome. Eligible for the study were female children with a confirmed pathogenic variant of the MECP2 gene and pharmacoresistant epilepsy at least two years old, with a minimum weight of 10 kg. Patients were treated with oral CBD solution as an adjunctive therapy. CBD was initially administered twice daily at a dose of 5 mg/kg/day; when no adverse effects were reported, the dose was increased by 5 mg/kg/day once a week, up to a maximum of 30 mg/kg/day. CBD reduced the incidence of seizures in 70% of patients, with one patient being seizure-free, two reducing seizures by more than 75%, and four reducing seizures by more than 50%. According to caregivers, 50% of patients showed a reduction in agitation or anxiety attacks. Improvement in spasticity was noted in 40% of patients. No aggravation of symptoms or side effects were observed. CBD has shown good activity in alleviating symptoms affecting patients with Rett syndrome [229]. When used as adjunctive therapy in the treatment of RTT patients with pharmacoresistant epilepsy, it is effective and well tolerated.

The mechanisms of cannabinoids in autism spectrum disorder are complex and not yet fully understood. However, research suggests several potential mechanisms through which cannabinoids, particularly CBD, may exert beneficial effects on the symptoms. Imbalances in the endocannabinoid system have been implicated in autism spectrum disorder [230,231,232]. CBD, by interacting with CB1 and CB2, and enzymes involved in endocannabinoid metabolism, may help restore balance and improve symptoms associated with autism spectrum disorder [233]. CBD has been shown to modulate the activity of various neurotransmitter systems implicated in autism spectrum disorder, including the serotoninergic and GABA-ergic systems [234,235,236,237]. Dysregulation of these neurotransmitter systems is commonly observed in individuals with ASD. By modulating neurotransmitter activity, CBD may help alleviate symptoms such as anxiety, depression, and hyperactivity [238,239].

#### 2.3.3. Tourette Syndrome

Tourette syndrome is a chronic neurodevelopmental disorder characterized by motor and phonic tics, which in turn can reduce the quality of life of affected individuals [240]. The prevalence of Tourette syndrome worldwide is 0.3% to 1%. In addition, 1% to 3% of children and adolescents may have a milder or unidentified form. Epidemiological studies have shown that 20% of children in the U.S. develop tics sometime in childhood, and the highest estimated prevalence of Tourette syndrome is probably 4–8 cases per 1000 children [241]. Treatment is complex, in part because of the variety of symptoms and comorbidities in individuals. The currently available therapeutic options are not sufficiently effective and do not change the long-term prognosis [208]. In addition, they can cause serious side effects such as parkinsonism, metabolic syndrome, and hyperprolactinemia.

Mueller-Vahn et al. performed a crossover study of 12 patients (18–66 years old) in 2002, which found that treatment with a single dose of THC was effective and safe in treating tics and obsessive-compulsive behavior [242]. Anis et al. [243] performed a clinical trial enrolling 18 patients aged 18 to 65. Three of the 18 patients (16.7%) terminated the study prematurely. The reasons for early treatment discontinuation were a severe depressive episode after recruitment before cannabis use in one patient, a presumed lack of efficacy and aversion to the smell of cannabis (withdrawal after the first visit), and worsening obsessive thinking and compulsions in one patient (withdrawal just before the second visit). The majority of patients (93%) who completed 12 weeks of cannabis use chose to consume MC through the lungs (smoking or inhaling). One patient (7%) used both sublingual oil and smoked cannabis. Another patient (7%) used the oil extract exclusively in sublingual administration. The study showed good efficacy and tolerability of medical marijuana in adult patients with Gilles de la Tourette syndrome as well as a subjective reduction in tics. The most common adverse event was dry mouth (67%), followed by fatigue (53%) and sedation and dizziness (47%). Three patients suffered from psychiatric side effects, including worsening of obsessive-compulsive disorder (treatment discontinuation), panic attacks, and anxiety (resolved after treatment modification). Six patients (40%) reported cognitive side effects related to time perception, visuospatial disorientation, confusion, slow processing speed, and attention. Abi-Jaoude et al., 2022, conducted a double-blind, randomized, controlled crossover trial of cannabis that examined the efficacy of single doses of three vaporized medical cannabis products and a placebo on tics in twelve adults with Tourette syndrome [244]. Nine participants completed the study. Participants received single doses (0.25 g) of three different cannabis medicinal products: THC 10%, CBD 9%/9%, CBD 13%, and placebo, administered at 2-week intervals. In terms of Tourette syndrome tics, there was no statistically significant difference for any of the cannabis products. However, the THC 10% product was significantly better than the placebo in secondary outcome measures. The 10% THC product caused the most adverse events. Reported adverse events after administration of the 10% THC product included sedation, psychomotor effects, dizziness, cough, burning throat, dry mouth, feeling cold, and feeling high. All three cannabis products caused reports of burning throat and dry mouth, while sedation, psychomotor effects, and dizziness were more common in the product containing 10% THC. An episode of fainting and seizures after administration of the 9%/9% THC/CBD product occurred in one participant, who subsequently fully recovered.

The CB2 receptor is expressed in brain regions involved in motor control, such as the substantia nigra and basal ganglia [245,246,247]. Activation of CB2 receptors inhibits the release of dopamine, a neurotransmitter implicated in motor function [245,248]. This inhibition of dopamine release by CB2 receptors helps regulate motor movements and reduce the frequency of repetitive behaviors. Activation of 5-HT receptors, specifically 5-HT2A/2C receptors, can induce repetitive behaviors [249]. Loss of functional CB2 receptors leads to increased repetitive behaviors following 5-HT2A/2C receptor activation, suggesting a regulatory role for CB2 receptors in this process [250]. Activation of 5-HT2A/2C receptors can modulate the expression levels of enzymes involved in endocannabinoid metabolism, such as ABHD6 and MAGL [250]. This modulation may lead to alterations in the levels of endocannabinoids like 2-AG and anandamide, which are implicated in Tourette syndrome [251]. Dysregulation of endocannabinoid levels, possibly induced by 5-HT2A/2C receptor activation, could contribute to the pathophysiology of Tourette syndrome. Activation of 5-HT2A/2C receptors may increase the expression of GPR55 [250]. The interplay between CB2 receptors and GPR55 suggests an even more complex regulatory network within the endocannabinoid system. 

### 2.4. Psychiatric Disorders

Currently, more than 260 million people worldwide suffer from anxiety and mood disorders, which affect an estimated 25% of the European population. In addition to their high prevalence, these mental disorders have a high incidence quotient, leading to a significant reduction in quality of life and interpersonal relationships [252]. 

Recent research shows three hypotheses surrounding the endocannabinoid system’s role in emotional and behavioral control [253]. The first is interaction with the stress response mechanism, notably the HPA-axis, which moderates behavioral reactions and maintains equilibrium [254]. Secondly, the integration of sensory perception with behavioral execution buffers against maladaptive responses and potentially protects against psychiatric symptoms [255]. Lastly, the promotion of proactive coping mechanisms in adversity fosters anxiolytic and antidepressant effects [256]. These hypotheses converge, highlighting the endocannabinoid system as a promising target for addressing affective dysregulation. 

#### 2.4.1. Anxiety

Anxiety disorders are divided into three categories: generalized anxiety disorder, paroxysmal anxiety syndrome, specific or social phobias, and social phobia [257]. Symptoms of the disorder include shortness of breath, inability to stay calm, sleep problems, feelings of anxiety, panic and fear, being cold and/or sweaty, heart palpitations, dry mouth, and nausea. 

The efficacy of CBD was assessed in patients previously diagnosed with anxiety disorders. In previously untreated patients with psychiatric disorders in social situations, oral THC 10 mg, CBD 600 mg, or a placebo was administered in three consecutive sessions, one month apart [258]. Physiological measurements and symptom assessments were assessed before 1, 2, and 3 h after drug administration. CBD, by inducing changes in regional brain blood flow, reduces subjective anxiety. A significant case series, including psychiatric patients whose primary problem was anxiety or sleep problems, suggests that administering CBD rapidly and persistently reduces distressing symptoms. CBD also improved sleep disorders within the first month of treatment but with fluctuations throughout the evaluated three-month period. However, Hundal et al. conducted a clinical study on 32 volunteers with high features of psychiatric disorders. CBD increased anxiety symptoms and had no effect on reality perception disorders. The individuals were assigned randomly to either receive oral CBD (600 mg) or a placebo 130 min prior to engaging in virtual reality. Established rating scales were employed to evaluate paranoid thoughts and anxiety. Throughout the experimental period, levels of salivary cortisol, heart rate, and blood pressure were monitored [259]. The results show that cannabinoids are effective in treating anxiety in patients with SAD, but do not have such an effect, for example, in patients with a high degree of paranoia, which is also accompanied by anxiety.

CBD interacts with various receptors in the central and peripheral nervous systems, including 5-HT1A, CB1, CB2, and TRPV1 receptors, which regulate fear and anxiety [260,261,262]. CBD’s acute anxiolytic effects at low to moderate doses involve 5-HT1A receptor activation, while higher doses exert anxiolytic effects through TRPV1 antagonism [263,264]. However, higher doses may also induce anxiety through TRPV1 agonism, a unique activity of CBD compared to THC [109]. CBD enhances endocannabinoid signaling indirectly by inhibiting the FAAH enzyme, which metabolizes anandamide, thereby increasing anandamide levels and CB1 receptor activation [110]. 

#### 2.4.2. Depression

Depression is described as a destructive mood regulation disorder. Patients suffering from depressive disorders experience emotional and cognitive changes, thoughts of death and suicide, loss of motivation, sleep disturbances, a constant sense of sadness, anxiety, guilt, irritability, impaired memory or appetite, fatigue, neglect of responsibilities, changes in personal life and withdrawal from others [265].

Symptoms of depression induced by various disorders were assessed across seven studies involving “dronabinol” compared to a placebo and one study compared to “Prochlorperazine”. In the case of “nabilone”, three studies comparing it to a placebo were conducted, along with two studies comparing it to the active drug. A placebo was pitted against CBD in six randomized controlled trials and also against nabiximols in seven RCTs. The comprehensive meta-analysis echoed the findings observed across all subgroups, indicating minimal to no alleviation of depressive symptoms. Regrettably, both CBD and nabilone showed no significant impact on depressive symptoms. However, dronabinol and nabiximols exhibited a slight improvement compared to the placebo, although the evidence was only moderately strong for nabiximols [266]. To date, no studies have been conducted regarding the primary outcome of depression. Three studies evaluating orally administered nabiximols for the treatment of other conditions (multiple sclerosis and cannabis withdrawal) did not show a significant impact on secondary depression outcomes [267,268]. It is worth noting that in one study involving cancer patients using nabiximols, significant mood reduction occurred in those using the highest dose (11–16 sprays per day) compared to the placebo. Furthermore, some epidemiological evidence has revealed a higher level of depressive symptoms in heavy cannabis users compared to light users and non-users [269]. Therefore, caution should be exercised with higher doses of THC in individuals suffering from major depressive disorder or low mood. However, a cross-sectional study on usage and perceived efficacy suggests that out of over 1429 participants identified as medical marijuana users, over 50% reported using medical marijuana specifically for treating depression [270].

#### 2.4.3. Post-Traumatic Stress Disorder

Post-traumatic stress disorder (PTSD) stems from exposure to severely traumatic events such as combat or interpersonal violence, leading to distressing symptoms, including intrusive memories, nightmares, and emotional withdrawal [271]. Co-occurring conditions, influenced by trauma exposure or shared causal determinants, exacerbate PTSD severity and disproportionately affect disadvantaged populations. Epidemiological studies reveal high rates of trauma exposure in both military personnel and civilians, with prevalence rates varying based on the level of trauma exposure. Certain traumatic events, such as rape or direct combat trauma, are associated with a significantly elevated risk for PTSD, with prevalence rates ranging from 25% to 50%. Despite the high frequency of trauma exposure, the majority of individuals do not develop PTSD, suggesting resilience factors at play. Cross-national differences in PTSD prevalence may be attributed to cultural variations in reporting or experiencing symptoms, while comorbid conditions such as depression and substance use disorders are strongly associated with PTSD, posing challenges for treatment [272].

A double-blind, placebo-controlled trial assessed the efficacy of nabilone in treating PTSD-related nightmares in male military personnel with chronic PTSD [273]. A study was conducted with a group comprising 10 Caucasian Canadian men aged between 18 and 65. The research involved the use of orally administered nabilone tablets, beginning at a dose of 0.5 mg and gradually increasing by 0.5 mg each week, up to a maximum dosage of 3.0 mg. Results revealed a significant reduction in CAPS Recurring and Distressing Dream Scores, with 70% of subjects in the nabilone group reporting improvement compared to 22% in the placebo group. Common side effects included dry mouth and headache, yet nabilone was generally well-tolerated, with no significant changes in vital signs observed [273]. Another randomized, double-blind, placebo-controlled crossover trial assessed smoked cannabis’s impact on PTSD symptoms in 80 US military veterans, with 76 completing Stage 1 and 74 proceeding to Stage 2 [274]. Cannabis samples included High THC, High CBD, THC + CBD, and placebo, with participants consuming up to 1.8 g/day for three weeks per stage, followed by a two-week cessation period. While cannabis usage averaged 8.4 to 14.6 g during Stage 1, with no significant differences between groups, the THC + CBD group consumed significantly more in Stage 2. Participants accurately guessed treatment assignments for High THC or THC + CBD, preferring THC + CBD treatment followed by High THC and High CBD. Common adverse events included cough, throat irritation, and anxiety, with rare serious events not requiring emergency unblindings. Moderate withdrawal symptoms decreased during treatment but increased post-cessation, particularly in the High THC group. Reductions in PTSD symptom severity were observed in all groups in Stage 1, with Stage 2 showing greater reductions in self-reported PTSD symptoms with THC + CBD compared to High CBD, and both THC + CBD and High THC leading to decreased depression and social anxiety [274]. Cannabinoids have been studied for their potential to alleviate sleep disorders in individuals by improving sleep patterns, enhancing general functioning, and enhancing overall quality of life. Studies have indicated that THC can facilitate shorter sleep latency, reduce difficulty falling asleep, and increase the time spent asleep [275,276]. Additionally, THC has been found to alleviate symptoms associated with sleep disorders such as sleep apnea by blocking serotonin-induced exacerbation [277]. At higher doses, THC-dominant cannabis decreases both the overall duration of rapid eye movement (REM) sleep and the density of REM activity [278]. Additionally, THC has been associated with improvements in sleep duration compared to CBD-dominant treatments [279].

### 2.5. Painful Pathological Conditions

#### 2.5.1. Migraine

Migraine presents itself through episodes of headaches that endure for 4–72 h. The pain is concentrated on one side, has a pulsating nature (the patient may sense a pulse in sync with their heartbeat, both during physical exertion and at rest), and ranges from moderate to substantial intensity [280]. It heightens during regular physical activities and is accompanied by symptoms such as nausea, sensitivity to light (photophobia), and sensitivity to sound (phonophobia). The etiology of migraines is believed to be multifactorial, involving a combination of genetic predisposition and environmental factors. Neurovascular reactions, triggered by cyclic changes in the central nervous system or specific triggers, contribute to the development of migraines [281]. While some individuals may experience only isolated episodes, a genetic threshold likely plays a role in the tendency for recurrent attacks [280]. Migraine is widespread, impacting approximately 12% of the population, with episodes occurring in about 17% of women and 6% of men annually [282].

The study of the effectiveness of using dried cannabis flowers administered by inhalation (pipe/vape) for the treatment of headache and migraine-related pain, conducted by Sarah S. Stith et al. incorporated data from 699 participants [283]. Patients were using the Releaf Application within the timeframe spanning from 10 June 2016 to 12 February 2019, encompassing a comprehensive analysis of 1910 session-level attempts devoted to ameliorating headache or migraine-related pain. The study assessed changes in pain intensity on a 0–10 VAS scale before and after cannabis use. Results showed that 94% of users felt relief within a two-hour window, and, on average, symptoms dropped by 3.3 points on a 0–10 VAS scale. The effectiveness of the treatment differed based on the percentage of THC and CBD content in the product, combustion methods (vape/pipe), and the age and gender of the patient. Regression analyses did not show significant differences in symptom relief by product characteristic (*C. sativa*/*C. indica*), except for a decrease associated with higher CBD products. Patients who were using pipes encountered a lower degree of relief than those who were using vaporizers. Interestingly, males experienced more relief than females. Younger individuals (patients under 35 years old) encountered reduced concluding symptom levels (*p* = 0.008), although this did not result in a statistically significant increase in symptom relief. Washington State University conducted a study with 1306 medical cannabis users, utilizing data from the Strainprint app, to assess the perceived effectiveness of inhaled cannabis, including methods like smoking and vaping, in treating headaches and migraines through 12,293 and 7441 monitored sessions, respectively [284]. The primary goal was to determine if inhaled cannabis reduced headache and migraine severity, with results showing a 50% reduction in migraine ratings. Predictors of these reductions, including gender, type of cannabis, CBD and THC content, and dose, were explored. The study acknowledged the development of tolerance with repeated cannabis use, yet it highlighted that cannabis did not lead to medication overuse headaches associated with conventional treatments. However, the text did not provide details on other potential side effects. The study suggests that inhaled cannabis reduces headache and migraine severity by around 50%, noting a potential risk of tolerance with repeated use. Importantly, cannabis use does not seem to result in medication overuse headaches, highlighting the need for future placebo-controlled trials to delve deeper into dose, cannabis type, and cannabinoid interactions. 

CBD reduces nitroglycerin-induced trigeminal hyperalgesia, with a reduction in gene expression levels of iNOS, CGRP, and pro-inflammatory cytokines in central and peripheral structures relevant to migraines experienced a notable decrease [285]. CBD attenuates hyperalgesia induced by carrageenan and nitroglycerine, suggesting its ability to modulate nociceptive signaling pathways [286]. This effect may involve the suppression of cyclooxygenase (COX) activity, prostaglandin production, and the overproduction of nitric oxide (NO), which are implicated in migraine pathophysiology [287]. CBD exhibits a non-linear dose response in migraine models, suggesting the presence of a ceiling effect [288]. CBD decreases nitroglycerine-induced IL-6 protein levels in migraine-relevant areas such as the medulla-pons and trigeminal ganglion [285]. Chronic CBD administration does not change FAAH gene expression induced by NTG administration [285,289]. This suggests that CBD’s anti-migraine effects may not be directly mediated by the inhibition of fatty acid amide hydrolase (FAAH), which is responsible for endocannabinoid breakdown. Instead, CBD may inhibit AEA reuptake, leading to increased levels of available endogenous cannabinoids [110]. CBD may interact with TRPV1 receptors, which are expressed in small trigeminal neurons involved in orofacial nociception [290]. 

#### 2.5.2. Neuropathic Pain

Neuropathic pain is described as pain caused by primary damage to the somatosensory part of the nervous system. The condition is continuous or recurrent, chronic, and is not limited by treatment of the underlying disease or the natural healing process. The cause can be a variety of neurological disorders affecting the central and peripheral nervous systems. Not every person experiencing a neurological disorder or injury develops neuropathic pain, suggesting that the onset of neuropathic pain can be a combination of clinical, genetic, biological, and psychosocial risk factors [291].

Dykukha et al. conducted a meta-analysis limited to randomized controlled trials with a double-blind design in patients with chronic neuropathic pain, primarily assessing pain reduction after administration of nabiximols or placebo [292]. Patients were treated with nabiximols as an oromucosal spray administered onto the oral mucosa for a period of 2 to 14 (average 7.2) weeks. The baseline pain severity was at least moderate, ranging from 4 points on a 0 to 10 NRS or BS 11 scale. The composite endpoint regarding changes in pain outcomes was significantly favorable for the nabiximols oromucosal spray compared to the placebo, eliciting a small but significant effect in all studies. Schimrigk et al. conducted a clinical trial on 240 patients aged 18–70 years with stable multiple sclerosis and moderate to severe central neuropathic pain [293]. The study included a 16-week randomized, double-blind, placebo-controlled phase followed by a 32-week open-label trial. Patients underwent a 4-week titration period to establish a tolerated dose of dronabinol, with dosage increases every 5 days. The pain reduction with dronabinol and placebo was similar during the treatment phase. Initially, adverse events were higher with dronabinol, but long-term therapy showed a decrease of 26%. No signs of drug abuse were observed, suggesting dronabinol is a safe option for long-term treatment of central neuropathic pain in multiple sclerosis. Wilsey et al. conducted a double-blind, placebo-controlled crossover study to assess the analgesic efficacy of vaporized cannabis in patients experiencing neuropathic pain resistant to conventional treatment [294]. Thirty-nine patients with central and peripheral neuropathic pain were subjected to a standardized inhalation procedure of either a moderate dose (3.53% THC), a low dose (1.29% THC) of cannabis, or a placebo using a Volcano vaporizer. The study considered pain intensity on a visual analog scale, psychoactive side effects, and neuropsychological outcomes. Participants attended three 6-h experimental sessions separated by at least 3 days to allow for the metabolic breakdown of THC metabolites. There was a 30% reduction in pain intensity compared to placebo. Side effects were minimal and manifested only as mild psychoactive effects and temporary, reversible neuropsychological effects. Indian hemp demonstrated analgesic efficacy, but the low dose reduced pain more than the moderate dose, which in turn reduced pain more than the placebo. The analgesic effect achieved with the low dose of THC (1.29%) in patients experiencing neuropathic pain despite conventional treatment methods is clinically significant.

Phytocannabinoids interact with the endocannabinoid system. THC acts as a CB_1_ and CB_2_ agonist [295]. Activation of these receptors can lead to the reduction of neuropathic pain signs such as allodynia and hyperalgesia [296]. CBD inhibits enzymes FAAH and MAGL responsible for endocannabinoid degradation, elevating endocannabinoid levels and leading to pain relief [109,297,298]. These inhibitors have shown efficacy in reducing allodynia in animal models of neuropathic pain [297,299,300,301]. THC reduces neurotransmitter release by neurons, especially glutamate, which plays a key role in pain signaling pathways [302]. Cannabinoids inhibit voltage-gated sodium and calcium channels (VGSCs and VGCCs), including specific subtypes such as Nav1.7–1.8 and CaV2.1–2 [303,304,305,306,307,308,309]. Moreover, cannabinoids act on ligand-gated ion channels such as TRPV1, TRPV2, glycine (GlyR), and GABA-A receptors [310,311]. THC acts as a TRPV2 agonist, while CBD acts as a TRPV1 agonist [113,312]. Modulation of these TRP channels affects pain sensation [313,314]. Activation of these channels, particularly TRPV1, can lead to analgesia. Additionally, cannabinoids like THC and CBD act as positive allosteric modulators of specific subtypes of these ion channels, further contributing to pain relief [311,315]. Cannabinoids target nuclear receptors such as PPAR-γ, which regulate gene expression and have been implicated in neuropathic pain [316,317]. Activation of PPAR-γ by cannabinoids mediates their anti-allodynic effects in neuropathic pain models [318,319,320,321]. In addition to cannabinoid receptors, cannabinoids also interact with other G protein-coupled receptors such as 5-HT1A and GPR55 [318,319,321,322]. Activation of these receptors, particularly 5-HT1A, has been shown to have anti-allodynic effects in neuropathic pain models [323,324]. THC inhibits COX-2, leading to increased levels of AEA and decreased levels of prostaglandins, which reduces inflammation and pain signaling [325].

#### 2.5.3. Diabetic Neuropathy

Diabetic neuropathy stands out as the most prevalent neurological complication of diabetes, showcasing subjective or clinical signs of damage to the peripheral nervous system, potentially affecting any part of it [326]. The primary clinical manifestation of diabetic polyneuropathy involves symmetrical pain in the limbs, particularly at the distal extremities, and the characteristic sensation is often likened to wearing gloves and socks [327]. Peripheral neuropathy is acknowledged as a chronic condition, persisting continuously or recurring episodically, generally unaffected by the natural healing process or the treatment of the underlying disease. Although the etiology remains partially understood, current knowledge suggests a multifaceted development of neuropathic pain, encompassing genetic, psychosocial, biological, and clinical factors. The escalating risk associated with diabetic neuropathy is directly linked to the prolonged duration of diabetes, and the number of individuals affected by this condition continues to rise. Projections for the year 2045 anticipate a global diabetic population reaching 629 million. Research indicates that among all diabetes patients, as many as 20–50% experience diabetic neuropathy [328].

In a study by Wallace et al., the efficacy of inhaled cannabis for painful diabetic peripheral neuropathy was investigated through a randomized, placebo-controlled, double-blind crossover trial involving 31 participants, of whom only 16 completed the study [329]. Participants aged 18 or older maintained stable glycemia (HbA1c ≤ 11%) with diabetes mellitus type 1 or type 2, and underwent four sessions separated by two weeks. Inclusion criteria required spontaneous and evoked foot pain and a minimum 6-month history of painful diabetic neuropathy. Subjects received varying THC-containing cannabis doses, with pain reduction as the primary outcome measured by a visual analog scale. Each participant received a placebo and a single dosing session with 1%, 4%, and 7% THC, with a two-week washout period to minimize carryover effects. Results indicated reductions in spontaneous and evoked pain scores for different THC doses, with the 7% THC dose showing the most effectiveness. The proportion of participants achieving at least a 30% pain reduction was higher with 4% and 7% THC doses compared to the placebo, but cognitive function worsened at the 7% THC dose. Adverse events included euphoria and sedation. A subsequent study by the same research team analyzed the relationship between plasma THC levels, pain outcomes, and cognitive function, including all sixteen participants from a previous study. Findings suggested a complex relationship between THC plasma levels and pain reduction, with an initial negative linear relationship followed by a positive linear relationship beyond a certain point. The concept of “self-titration” was introduced to account for individuals’ adjustments in inhalation patterns based on THC tolerance. The study emphasized the importance of measuring cannabinoid plasma levels for accurate therapeutic assessment, highlighting the significance of actual inhaled doses and serum concentrations. Minimal cognitive effects were observed at low plasma THC levels during pain relief. However, limitations such as acute dosing in cannabis-naive individuals and small sample size were acknowledged, while the crossover design was praised for enhancing statistical power [330]. Bagher et al. studied the impact of the endocannabinoid system on diabetic neuropathic pain and primarily used mice with streptozotocin-induced diabetes [331]. Alterations in CB1 and CB2 receptor expression and endocannabinoid levels across various areas of the nervous system in mice with diabetic neuropathy. Preclinical studies hint at the potential effectiveness of CB1 receptor agonists, CB2 receptor agonists, and endocannabinoid-metabolizing enzyme inhibitors in alleviating diabetic neuropathic pain. Nevertheless, further clinical studies are necessary, considering species differences, to fully understand and evaluate potential therapies based on the endocannabinoid system. 

#### 2.5.4. Fibromyalgia

Fibromyalgia is a rheumatic disorder affecting soft tissues, characterized by chronic, generalized musculoskeletal pain and tenderness at typical tender points [332,333]. Sleep disturbances, chronic fatigue, exercise intolerance, and psychopathological symptoms accompany it. The disease affects 2–4% of the general population, predominates in individuals of the White race, and is more common in women (6–10 times more frequently). Typically, the disease affects individuals in their 3rd to 5th decade of life. The etiology of the disease is still debated and remains inconclusive. It is characteristic for the onset of symptoms to be associated with various stressors, both endogenous (mainly endocrine disorders, inflammatory rheumatic diseases, and psychiatric disorders) and exogenous (social problems, injuries, overloads, and weather changes). 

Van de Donk et al. conducted an experimental, randomized, placebo-controlled, 4-way crossover study to gather information on the analgesic effects of pharmaceutical-grade inhaled marihuana [334]. The study involved 20 patients (39 +/− 13 years old) with chronic fibromyalgia pain, who were tested with four different strains of cannabis administered by inhalation: Bedrocan (22.4 mg THC, <1 mg CBD), Bediol (13.4 mg THC, 17.8 mg CBD), Bedrolite (18.4 mg CBD, <1 mg THC), and a placebo strain without THC or CBD. During the active cannabis therapy, adverse effects such as inhalation-related cough, throat pain, an unpleasant taste, drug high, dizziness, and nausea were reported. None of the cannabis strains showed superior pain relief to the placebo, but Bediol treatment resulted in a 30% reduction in pain outcomes compared to the placebo. THC-containing strains increased the pressure pain threshold, while CBD inhalation increased THC serum concentration but reduced THC’s analgesic effect. Chaves et al. undertook a study to determine the benefits of using THC-rich cannabis oil in patients with fibromyalgia [335]. The study included 17 female patients (8 cannabis, 9 placebo) with a mean age of 52 years, conducted over eight weeks involving the administration of cannabis oil (24.44 mg/mL THC and 0.51 mg/mL CBD). Initially, a dose of 1 drop per day (1.22 mg THC and 0.02 mg CBD) was applied, with the possibility of dose escalation during the study based on symptoms, assessed every ten days. The cannabis group reported drowsiness, dizziness, dry mouth, mood, and libido improvement with an average dose of 3.6 drops/day. The placebo group reported one case of drowsiness with 4.3 drops/day of olive oil. After the intervention, the cannabis group showed a significant decrease in the Fibromyalgia Impact Questionnaire score compared to the placebo. THC-rich cannabis oil improved well-being, reduced pain and fatigue, and enhanced work quality. No serious adverse effects were observed, indicating phytocannabinoids as a safe and accessible therapy for fibromyalgia. Ware et al. conducted a study to assess the effectiveness of nabilone in treating sleep disorders in fibromyalgia [336]. The study included 29 patients with fibromyalgia and chronic insomnia who underwent treatment with two medications administered for 2 weeks with a 2-week break between them. The drugs compared were nabilone at a dose of 0.5–1.0 mg before bedtime and amitriptyline 10–20 mg before bedtime. The results showed that both drugs improved sleep, but nabilone was found to be superior (Insomnia Severity Index difference = 3.2). Nabilone was less effective during awakening (difference = 0.3), while it performed better during rest (Leeds Sleep Evaluation Questionnaire difference = 0.5). Only mild to moderate adverse effects were noted, mainly related to nabilone (nausea, dizziness, dry mouth).

Although clinical evidence for cannabinoid treatment of fibromyalgia is limited, there is substantial preclinical data suggesting a molecular foundation for the analgesic properties of cannabinoids. However, the molecular mechanisms of cannabinoids in fibromyalgia are still being investigated. Cannabinoids are believed to exert their effects on fibromyalgia through pain modulation as cannabinoid receptors CB1 and CB2 impact nociception, reducing the sensation of pain [337,338]. Chronic pain conditions like fibromyalgia often involve sensitization of nociceptive sensory pathways. Cannabinoids may help reduce this sensitization, thereby alleviating pain in fibromyalgia patients [339]. Cannabinoids have been shown to exert immunomodulatory effects [340,341,342]. By modulating the immune response, cannabinoids could potentially help alleviate symptoms associated with inflammation in fibromyalgia.

#### 2.5.5. Trigeminal Neuralgia

Trigeminal neuralgia’s etiology is often associated with compression or irritation of the trigeminal nerve, a key facial nerve responsible for sensations in the face. This compression can be caused by blood vessels, tumors, or other structural abnormalities, leading to intense, stabbing facial pain [343,344]. It is characterized by recurring short-lasting (from <1 s to 2 min) episodes of intense, unilateral facial pain resembling an electric shock, within the innervation area of the trigeminal nerve. The prevalence of trigeminal neuralgia in the population is estimated to be 0.16–0.3% over a lifetime. The distribution showed a female predominance, with the highest prevalence in the age group of 51–59 years. The annual incidence ranges from 4 to 29 new cases per 100,000 people [345].

The study conducted in Buffalo, New York, aimed to assess the efficacy and adverse effects of medical cannabis in treating trigeminal neuralgia ([346] The study included 42 patients with trigeminal neuralgia (32 female, 10 male) who were treated with medical cannabis through the New York State Medical Marijuana Program. Patients were using sublingual drops as the route of administration for cannabinoid therapy. In Mechtler et al.’s study, 81% of the 42 patients reported improvement in their trigeminal neuralgia symptoms. Of the patients reporting ≥50% improvement, 69% used one product, and 50% used a 1:1 THC to CBD ratio. Moreover, 50% of patients initially using opioids were able to reduce opioid consumption with medical cannabis. Patients reporting ≥50% improvement in trigeminal neuralgia symptoms often used a 1:1 ratio of THC to CBD. Of those initially using opioids, 50% were able to reduce opioid consumption with medical cannabis. Adverse effects of cannabinoid therapy were reported by 40% of patients, including fatigue, somnolence, nausea, and dizziness. Additionally, the text mentions common side effects of CBD, such as dry mouth, dizziness, drowsiness, fatigue, reduced appetite, nausea, vomiting, and diarrhea. The authors discuss potential drug interactions, including the inhibitory effects of CBD on certain cytochrome P450 enzymes. It highlights interactions with prednisolone, hydrocortisone, naproxen, tramadol, amitriptyline, gabapentin, pregabalin, and certain antidepressants [346].

A summary of the use of cannabis and cannabis-derived products in treating neurological diseases of various etiologies was presented in Table 1.

## 3. Next Steps in the Research Field

Research on cannabis in neurology has made significant strides in recent years, but there are still many avenues to explore (Table 2). Addressing these research gaps will not only advance our understanding of the therapeutic potential of cannabis but also guide the development of safe and effective treatments for patients with neurological disorders.

## 4. Conclusions

Cannabis and its active compounds have shown promise in relieving symptoms associated with various neurological disorders of different etiologies. Studies suggest their potential efficacy in managing conditions like epilepsy, multiple sclerosis, Parkinson’s disease, and neuropathic pain, among others. The neuroprotective properties of cannabinoids have been explored in research, indicating their ability to mitigate inflammation, oxidative stress, and neuronal damage. Cannabis-based medications have been approved in some regions for treating certain neurological conditions, demonstrating their therapeutic potential. However, individual responses to cannabis rich in THC can vary significantly, with some experiencing adverse effects such as anxiety, paranoia, and cognitive impairment. Healthcare professionals should carefully assess the potential benefits and risks of cannabis therapy on a case-by-case basis, considering factors such as individual medical history, concurrent medications, and lifestyle factors.

## Figures and Tables

**Table 1:** The use of cannabis, cannabis-derived products in the treatment of neurological diseases of various etiologies (Abbrev.—cannabidiol (CBD), tetrahydrocannabinol (THC)).

Disease	Preparation	Study	Sample Size	Outcomes	Observed Adverse Events
Epilepsy	CBD-enriched medical cannabis	Tzadok et al. [99]	74 patients	- 89% reduction in seizure frequency - Improvement in behavior in 44% of patients	Seizure aggravation, somnolence/fatigue
Epilepsy	Artisanal CBD preparations	Porcari et al. [100]	209 patients	- Reduction in seizure frequency of at least 50% observed in 33% of the CBD group, 44% of the CBD + clobazam group, and 38% of the clobazam group - Minimal differences between groups suggested that CBD may provide comparable benefits regardless of clobazam use	Sedation (more frequent in the clobazam group)
Epilepsy	CBD-based products	Pamplona et al. [101]	670 patients	- CBD significantly reduced seizure frequency, with 64% of patients showing improvement - Studies with CBD-rich cannabis extracts (71%) reported more improvement than those with purified CBD (46%) - Secondary health improvements were reported in 52% of patients, including improvements in awareness, sleep quality, mood, behavior, language, and motor skills	Appetite alteration, sleepiness, gastrointestinal disturbances, weight changes, fatigue, nausea
Dravet syndrome	CBD oral solution	Devinsky et al. [104]	120 patients	- Resulted in a more significant reduction in convulsive seizure frequency than placebo - Patients who became seizure-free was 5% with CBD	Diarrhea, vomiting, fatigue, pyrexia, somnolence, abnormal results on liver-function tests
Alzheimer’s disease	Nabilone	Herrmann et al. [158]	39 patients	- Nabilone showed efficacy in reducing agitation with a medium effect size	Sedation
Dementia	Oral THC	van den Elsen et al. [159]	22 patients	- Significant increases in dynamic balance, stride length, and gait velocity after THC administration compared to placebo	Dizziness, somnolence, balance disorders, and falls
Parkinson’s disease	CBD/THC product	Sousa et al. [130]	15 patients	- Pain reduction, anxiety relief, and improved sleep quality	Exhibited poorer cognitive ability
Multiple sclerosis	Synthetic or herbal and plant-derived cannabinoids	Haddad et al. [176]	3763 patients	- Reduction in the severity of multiple sclerosis-induced spasticity	Not found
Multiple sclerosis	Medical cannabis	Rainka et al. [177]	141 patients	- 72% experienced pain relief, 48% reported reduced muscle spasticity, and 40% improved sleep. Lesser improvements were noted in gait (11%), anxiety (11%), and quality of life (7%)	Not found
Multiple sclerosis	Extract of cannabis	Zajicek et al. [178]	279 patients	- Degree of muscle stiffness decreased	Not found
Amyotrophic lateral sclerosis	Nabiximols	Riva et al. [194]	59 patients	- 0.11 improvement on the Modified Ashworth scale	Not found
Autism spectrum disorder	Oral drops of CBD oil	Barchel et al. [206]	53 patients	- Reduction in aggressive behavior and self-injury, hyperarousal, and sleep symptoms improved	Drowsiness and loss of appetite
Autism Spectrum Disorder	CBD-rich cannabis	Aran et al. [223]	60 patients	- Improvements in aggressive behavior, anxiety	Sleep disturbances, loss of appetite, and irritability
Autism Spectrum Disorder	CBD-Enriched *Cannabis sativa* extract	Fleury-Teixeira et al. [224]	18 patients	- Sleep and behavioral disorders, as well as in motor development, communication, social interaction, and cognitive performance	Drowsiness, irritability, diarrhea, increased appetite, conjunctival congestion, and hyperthermia
Autism Spectrum Disorder	Cannabis oil containing 30% CBD and 1.5% THC	Bar-lev Schleider et al. [225]	188 patients	- Significant improvement in Autism Spectrum Disorder	Drowsiness, psychoactive symptoms, increased appetite, digestive disturbances, dry mouth, lack of appetite
Rett syndrome	Epidyolex^®^, CBD, 100 mg/mL oral solution	Desnous et al. [229]	46 patients	- Reduced the incidence of seizures, a reduction in agitation or anxiety attacks, improvement in spasticity	No aggravation of symptoms or side effects was observed
Tourette syndrome	Medical cannabis	Anis et al. [243]	18 patients	- Subjective reduction in tics	Dry mouth, followed by fatigue, sedation, and dizziness; three patients suffered from psychiatric side effects, including worsening obsessive-compulsive disorder (treatment discontinuation), panic attacks, anxiety, visuospatial disorientation, confusion, slow processing speed, and attention
Tourette Syndrome	vaporized 0.25 g dose of THC 10%, THC/CBD 9%/9%, CBD 13%	Abi-Jaoude et al. [244]	12 patients	- In terms of Tourette syndrome tics, there was no statistically significant difference for any of the cannabis products	Sedation, psychomotor effects, dizziness, cough, burning throat, dry mouth, feeling cold and feeling high, burning throat and dry mouth, while sedation, psychomotor effects, and dizziness
Attention Deficit-Hyperactivity Disorder	Sativex, oromucosal spray	Cooper et al. [211]	30 patients	- Reduction of symptoms and no cognitive impairments following cannabinoid use	Light-headedness and diarrhea, one participant taking the active medication experienced sudden onset of muscular seizures/spasms
Attention Deficit-Hyperactivity Disorder	Cannabis	Rasmussen et al. [212]	88 patients	- Subcortical regions like the right caudate exhibited reduced activation, an interaction - The effect between ADHD and cannabis was observed in the right hippocampus and - Cerebellar vermis, cannabis use did not impact behavioral response inhibition	Not found
Anxiety	CBD	Shannon et al. [258]	72 patients	- Decrease anxiety or sleep problems, reduce distressing symptoms,	Not found
Anxiety	CBD	Hundal et al. [259]	32 patients	- Effective in treating anxiety	Not found
Post-traumatic stress disorder	Nabilone	Jetly et al. [273]	Ten patients	- The results revealed a significant reduction in CAPS (Clinician-Administered PTSD Scale) Recurring and Distressing Dream Scores among the participants who received nabilone compared to those who received a placebo	Dry mouth, headache
Post-traumatic Stress Disorder	Cannabis	Bonn-Miller et al. [274]	76 patients	- Both THC + CBD and High THC led to decreased depression and social anxiety	Cough, throat irritation, anxiety
Neuropathic pain	Dronabinol	Schimrigk et al. [293]	240 patients	- Pain intensity during 16 weeks of dronabinol and placebo treatment was reduced by 1.92 and 1.81 - No signs of drug abuse and only one possible case of dependency occurred	Restlessness, irritability, sleep interference, decreased appetite, excessive sweating
Neuropathic pain	Cannabis	Wilsey et al. [294]	39 patients	- Pain relief appears to be maximal after the second dosing at 180 min postbaseline, but the peak effect drops off 1 to 2 h later - Cannabis has analgesic efficacy, with the low dose being as effective a pain reliever as the medium dose	There were no study-related serious adverse events
Fibromyalgia	Cannabis	Van de Donk et al. [334]	20 patients	- Cannabis varieties containing THC caused a significant increase in pressure pain threshold relative to placebo	Deterioration in mood and alertness, sore throat and sour taste, coughed during inhalation, nausea without vomiting
Fibromyalgia	THC-rich cannabis oil	Chaves et al. [335]	17 patients	- Group presented significant improvement on the “feel good”, “pain”, “do work”, and “fatigue” scores on the FIQ - THC-rich cannabis oil improved well-being, reduced pain and fatigue, and enhanced work quality	Somnolence, dizziness, mouth dryness, improved mood, improved libido
Fibromyalgia	Nabilone	Ware et al. [336]	29 patients	- Nabilone improved sleep - Nabilone was less effective during awakening, while it performed better during rest	Nausea, dizziness, dry mouth
Trigeminal neuralgia	Medical cannabis	Mechtler et al. [346]	42 patients	>50% improvement in reducing trigeminal neuralgia symptoms, 50% of patients were able to reduce opioids	Fatigue, reducing appetite, nausea, vomiting, diarrhea
Migraine	Cannabis	Stith et al. [283]	669 patients	- 94% of users felt relief. Symptoms dropped 3.3 points on the 1–10 vas scale	Not found
Migraine	Cannabis	Cuttler et al. [284]	1306 patients	- 56% reduction of headache and migraine symptoms	Not found
Diabetic neuropathy	Cannabis	Wallace et al. [329]	16 patients	- Reduction in spontaneous and evoked pain scores - Cognitive function worsening at the 7% THC dose	Euphoria, sedation
Diabetic Peripheral Neuropathy	1%, 4%, or 7% THC dose of cannabis	Wallace et al. [330]	16 patients	- Minimal cognitive effects observed at low plasma THC levels during pain relief	Not found

**Table 2:** Concerns and next steps in research in the field.

Concerns	Explanation
Clinical trials with specific cannabinoids	There is a lot of research on Δ^9^-THC and CBD. However, other cannabinoids have not been extensively studied. Future research could focus on clinical trials to investigate the therapeutic potential of these lesser-known cannabinoids and their derivatives, which have proven in vitro or in vivo biological activity, such as the following: THCV—antiepileptic activity [347], potential against Parkinson’s disease [348,349], antipsychotic effects [350], CBG—anticonvulsant activity [351], counteract neuroinflammation [352], neuroprotective in Huntington’s disease [202], in multiple sclerosis [353], CBG derivatives—anti-inflammatory in multiple sclerosis [354], in amyotrophic lateral sclerosis [355], neuroprotective potential in Huntington’s disease [356], in Parkinson’s disease [357,358,359], CBC—promotes neural stem/progenitor cells viability [360], induces neuronal differentiation in NSC-34 Cells [361], antinociceptive activity [362], anti-inflammatory [363], CBN—neuroprotective activity [167], antiseizure potential [364].
Long-term side effects	Cannabis use can have both short-term and long-term adverse effects, influenced by factors like frequency, THC strength, age, and individual susceptibility. Prolonged use may lead to cognitive decline, especially in younger patients, while also increasing the risk of psychiatric disorders and addiction [365,366,367]. However, understanding the long-term effects of medicinal cannabis remains limited. A meta-analysis found long-term medicinal cannabis use safe for chronic non-cancer pain (12 months) [368]. Still, research results on patients with multiple sclerosis vary, with some indicating potential benefits for cognition while others warn of increased psychiatric risks [176,369]. More research is needed to clarify the long-term safety and efficacy of medical cannabis.
Mechanisms of action	Although we have some understanding of how cannabinoids interact with the endocannabinoid system and other neurotransmitter systems in the brain [370,371,372,373,374,375,376], further research is needed to elucidate the precise mechanisms of action underlying the therapeutic effects of cannabis in neurological disorders.
Individual responses to cannabis	The delivery route, individual traits (age, sex), and genetic predispositions might influence the responses of patients to cannabis [377,378,379,380,381,382,383,384]. Future research should explore genetic, epigenetic, and pharmacogenomic factors to understand individual variability in cannabis response better and personalize treatments accordingly.
Clinical guidelines and education	Standardized dosing for cannabis-based treatments remains elusive. Establishing a consensus and clear clinical guidelines for the use of cannabis in neurological disorders is essential [385]. More research is imperative to develop evidence-based guidelines for healthcare providers; there also is a need for enhanced education among healthcare professionals and patients alike regarding the potential benefits and risks of cannabis-based treatments [386,387,388,389,390].
